# The anti-inflammatory effect of dimethyl trisulfide in experimental acute pancreatitis

**DOI:** 10.1038/s41598-023-43692-9

**Published:** 2023-10-05

**Authors:** Erik Márk Orján, Eszter Sára Kormányos, Gabriella Mihalekné Fűr, Ágnes Dombi, Emese Réka Bálint, Zsolt Balla, Beáta Adél Balog, Ágnes Dágó, Ahmad Totonji, Zoárd István Bátai, Eszter Petra Jurányi, Tamás Ditrói, Ammar Al-Omari, Gábor Pozsgai, Viktória Kormos, Péter Nagy, Erika Pintér, Zoltán Rakonczay, Lóránd Kiss

**Affiliations:** 1 Department of Pathophysiology, University of Szeged, Semmelweis U. 1, 6725 Szeged, Hungary; 2 Department of Pharmacology and Pharmacotherapy, Medical School, University of Pécs, Pécs, Hungary; 3 Department of Molecular Immunology and Toxicology and the National Tumor Biology Laboratory, National Institute of Oncology, Budapest, Hungary; 4 Doctoral School of Molecular Medicine, Semmelweis University, Budapest, Hungary; 5 Department of Anatomy and Histology, ELKH Laboratory of Redox Biology, University of Veterinary Medicine, Budapest, Hungary; 6 Chemistry Institute, University of Debrecen, Debrecen, Hungary

**Keywords:** Acute pancreatitis, Pharmacology

## Abstract

Various organosulfur compounds, such as dimethyl trisulfide (DMTS), display anti-inflammatory properties. We aimed to examine the effects of DMTS on acute pancreatitis (AP) and its mechanism of action in both in vivo and in vitro studies. AP was induced in FVB/n mice or Wistar rats by caerulein, ethanol-palmitoleic acid, or L-ornithine-HCl. DMTS treatments were administered subcutaneously. AP severity was assessed by pancreatic histological scoring, pancreatic water content, and myeloperoxidase activity measurements. The behaviour of animals was followed. Pancreatic heat shock protein 72 (HSP72) expression, sulfide, and protein persulfidation were measured. In vitro acinar viability, intracellular Ca^2+^ concentration, and reactive oxygen species production were determined. DMTS dose-dependently decreased the severity of AP. It declined the pancreatic infiltration of leukocytes and cellular damage in mice. DMTS upregulated the HSP72 expression during AP and elevated serum sulfide and low molecular weight persulfide levels. DMTS exhibited cytoprotection against hydrogen peroxide and AP-inducing agents. It has antioxidant properties and modulates physiological but not pathophysiological Ca^2+^ signalling. Generally, DMTS ameliorated AP severity and protected pancreatic acinar cells. Our findings indicate that DMTS is a sulfur donor with anti-inflammatory and antioxidant effects, and organosulfur compounds require further investigation into this potentially lethal disease.

## Introduction

Acute pancreatitis (AP) is an illness of sudden pancreas inflammation. It is a relatively prevalent condition among gastrointestinal disorders, and its occurrence is rising over time^1,2^.The disease severity can be categorised as mild, moderately severe, and severe groups following the Revised Atlanta Classification system, which is determined by the presence and duration of organ failure^3^. The overall mortality of AP is ~ 2%, but in severe cases, it can reach 30%^4^. Massive alcohol consumption, biliary obstructions, hypertriglyceridemia and other factors can initiate the disease. The pathomechanism of AP comprises pathological Ca^2+^ signalling, which induces nuclear factor κB (NF-κB) translocation into the nucleus inducing the expression of pro-inflammatory cytokines^5–7^. Cytokine release causes leukocyte recruitment, specifically neutrophils will migrate to the damaged site, and release enzymes (e.g. myeloperoxidase–MPO), cytokines and reactive oxygen species (ROS). These then aggravate the disease progression^8^. The Ca^2+^ signals also initiate early intra-acinar activation of digestive enzymes. This process will result in the self-digestion of the tissue. Additionally, intra-acinar generation of ROS, mitochondrial damage, impaired autophagy, and endoplasmic reticulum stress are also significant cellular processes during AP^7^. Although many aspects of the disease pathomechanism are identified, unfortunately, it is not fully understood and there is no specific treatment for AP^9^.

Sulfur-containing organic agents are called organosulfur molecules. Various members are naturally occurring in plants (garlic, onion, broccoli), food (cheese), or carcasses, but there exist synthetic ones as well. Many of them are biologically active compounds and also potential hydrogen sulfide (H_2_S) donors^10^. Their anti-inflammatory and cytoprotective effects have been described in several disease conditions^11,12^. Organosulfur molecules could affect certain signalling pathways (NF-κB, nuclear factor-erythroid 2 related factor 2–Nrf2, Phosphoinositide 3-kinase/Protein kinase B etc.), influence protein function via persulfidation, activate ion channels (K^+^, Ca^2+^, transient receptor potential ankyrin–TRPA, or transient receptor potential vanilloid–TRPV channels), and exhibit antioxidant properties, which all can contribute to their biological activity^13^. Additionally, these effects may be linked to H_2_S release from these molecules. H_2_S is a small signalling molecule, and its actions are heavily investigated in many diseases. In general, upregulation of H_2_S synthesis or administration have protective effects in inflammatory conditions^14^, while in AP its overexpression can exacerbate the inflammation through NF-κB activation and substance-P release^15^. Remarkably, some organosulfur molecules have already been tested in AP. Diallyl disulfide, a naturally occurring organosulfur, lowered the mRNA expressions of cystathionine-γ-lyase enzyme (CSE, one of the enzymes responsible for H_2_S synthesis), neurokinin-1-receptor, preprotachykinin A, and tumour necrosis factor-α (TNF-α)^16^. It reduced the synthesis of endogenous H_2_S in AP^17^ and led to reduced inflammation. Treatments with S-diclofenac and S-propargyl-cysteine organosulfur molecules slightly decreased the severity of AP in mice as well^18,19^. Based on these controversial observations we considered that it would be beneficial to test the effects of additional organosulfur molecules in this disorder.

Dimethyl trisulfide (DMTS) is a member of naturally occurring organosulfurs. It reacts with haemoglobin and produces methaemoglobin^20^, initiates the TRPA1 ion channel, and modulates pain sensation^21^, it has a great potential to decrease cyanide poisoning^22^, and reduces inflammation in serum-transfer arthritis^23^. Nevertheless, there is no literature regarding their effect on AP. We aimed to examine how DMTS influences the severity of AP and to provide insights into their mechanisms of action in both in vivo and in vitro studies.

## Results

### DMTS reduces the severity of acute pancreatitis in both mice and rats

**Figure 1:**
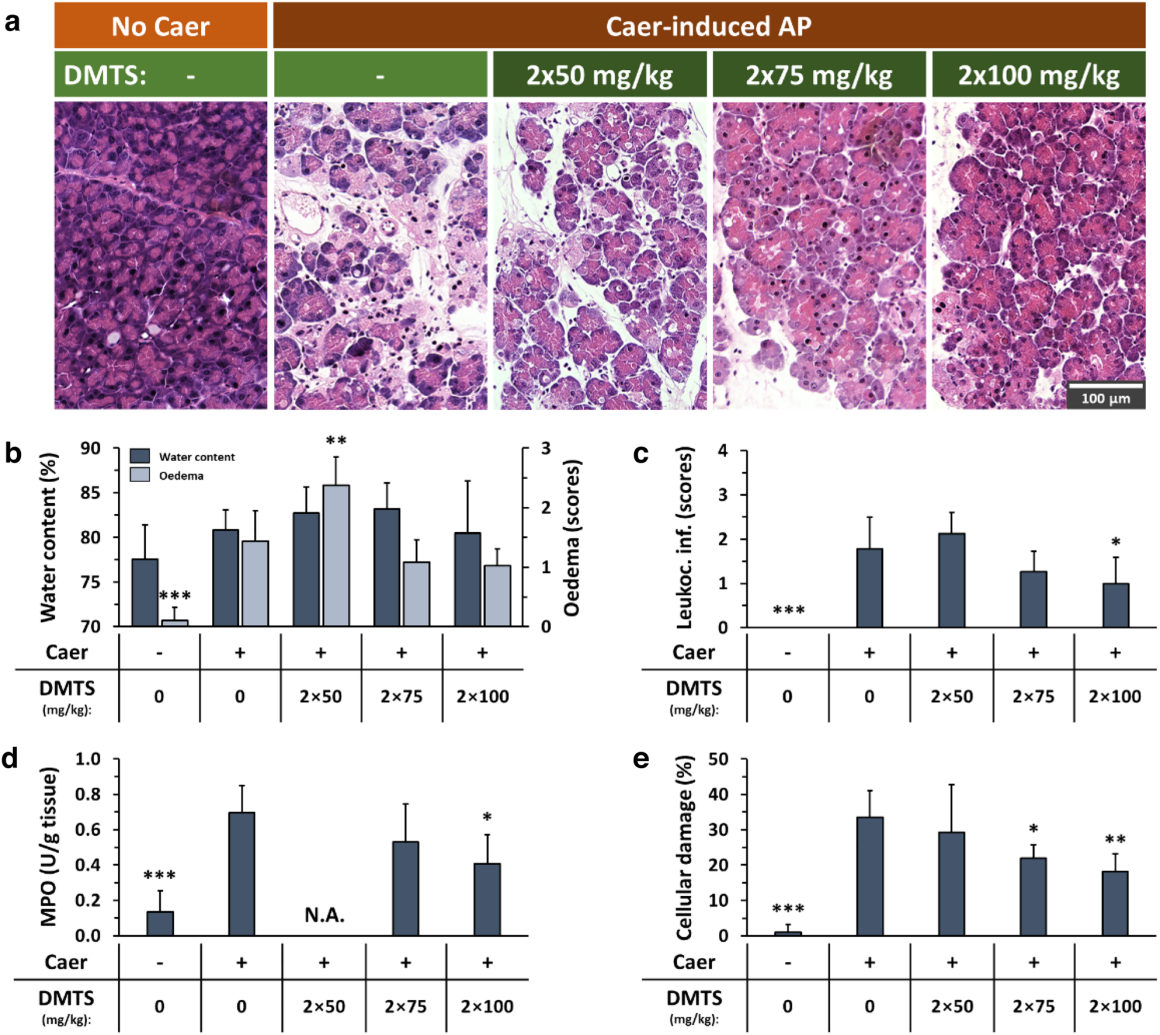
Dimethyl trisulfide (DMTS) administration lowers the severity of caerulein (Caer)-induced necrotizing acute pancreatitis (AP). Mice were treated subcutaneously with 2 × 50, 2 × 75 or 2 × 100 mg/kg DMTS, whereas intraperitoneal injection with 10 × 50 µg/kg Caer was used to induce AP. Control animals received physiological saline rather than Caer, or vehicle instead of DMTS. Then, at 12 h after the first Caer or physiological saline injection, animals were sacrificed. (**a**) Representative histopathological images of pancreatic tissues of the treatment groups. Bar charts demonstrate the extent of pancreatic (**b**) water content (as measured by the dry–wet weight ratio) and oedema (evaluation of histological sections), (**c**) leukocyte infiltration, (**d**) myeloperoxidase (MPO) activity, and (**e**) cellular damage. Values represent means with standard deviation (SD). The total number of animals was 42, for details of exact means, SDs, and animals per group please view Supplementary Table 1. (**b**–**d**) One-way ANOVA was carried out followed by Dunnett’s post-hoc test where all of the groups were compared to the Caer-only group, *p < 0.05; **p < 0.01; ***p < 0.001. (**e**) Kruskal–Wallis test was performed followed by Dunn’s post-hoc test, the groups were compared to the Caer-only group, *p < 0.05; **p < 0.01; ***p < 0.001. *Leukoc. inf*., leukocyte infiltration.

In vivo AP models of mice and rats were employed to examine the effect of subcutaneously (s.c.) administered DMTS. The pancreata of the control group possessed normal morphology (Fig. 1a) and DMTS treatment alone did not induce any visible alterations in the tissue (Fig. S1). AP caused by supramaximal doses of caerulein (Caer) resulted in elevated pancreatic oedema, intensive leukocyte infiltration, and tissue MPO activity, as well as 30–40% tissue damage (Fig. 1). The administration of DMTS showed a dose-dependent effect. 2 × 50 mg/kg DMTS had no effect on the disease, 2 × 75 mg/kg DMTS treatment significantly lowered pancreatic injury in AP mice (Fig. 1e), but this dose did not affect other measured parameters, and the highest dose of DMTS (2 × 100 mg/kg) significantly decreased pancreatic leukocyte infiltration, MPO activity, and the tissue damage as well.

Following prior positive results with two times DMTS administrations, a rapid test with other DMTS doses and treatment arrangement was conducted in the same Caer-induced AP model (Fig. S2). DMTS was administered four times at 50 and 75 mg/kg doses, and these interventions alone did not lead to any significant changes compared to the control (Fig. S3). Representative images demonstrate the result of the treatments on the pancreas morphology (Fig. S2A). DMTS had no effect on tissue water content during the disease and comparable findings were found with oedema scores (Fig. S2B). Four doses of DMTS, in contrast, dramatically reduced the tissue damage in AP (Fig. S2D), and this impact was independent of leukocyte infiltration (Fig. S2C).

**Figure 2:**
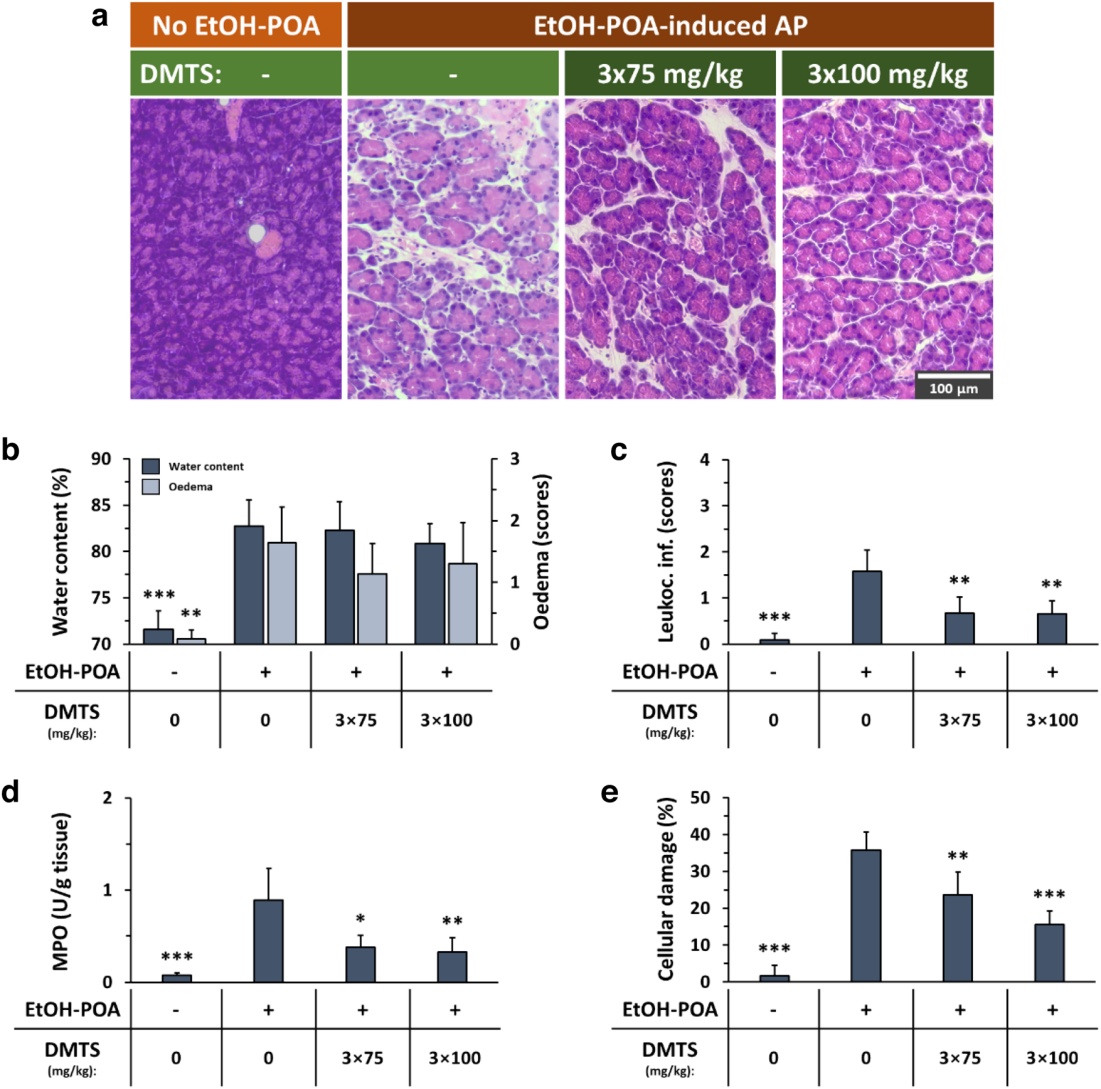
Dimethyl trisulfide (DMTS) administration reduces the severity of ethanol and palmitoleic acid (EtOH-POA)-induced necrotizing acute pancreatitis (AP). Mice were treated subcutaneously with 3 × 75 or 3 × 100 mg/kg DMTS, whereas intraperitoneal injection with 2 × 1.35 g/kg EtOH and 2 × 150 mg/kg POA were used to induce AP. Control animals were given physiological saline instead of EtOH-POA, or vehicle rather than DMTS. Animals were sacrificed 24 h after the first injection of EtOH-POA or physiological saline injection. (**a**) Representative histopathological images of pancreatic tissues of the treatment groups. Bar charts illustrate the extent of pancreatic (**b**) water content (as measured by the dry–wet weight ratio) and oedema (evaluation of histological sections), (**c**) leukocyte infiltration, (**d**) myeloperoxidase (MPO) activity, and (**e**) cellular damage. Values represent means with standard deviation (SD). The total number of animals was 25, for details of exact means, SDs, and animals per group please view Supplementary Table 1. (**b**, **e**) One-way ANOVA was carried out followed by Dunnett’s post-hoc test where all of the groups were compared to the EtOH-POA only group, **p < 0.01; ***p < 0.001. (**c**, **d**) Kruskal–Wallis test was conducted followed by Dunn’s post-hoc test, the groups were compared to the EtOH-POA only group, *p < 0.05; **p < 0.01; ***p < 0.001.

Another model of necrotizing AP induced with ethanol and palmitoleic acid treatment (EtOH-POA) was used in mice to test the effect of DMTS (Fig. 2). The treatment doses of DMTS of 3 × 75 and 3 × 100 mg/kg were given 1, 6, and 12 h following the onset of the inflammation. The EtOH-POA AP model increased pancreatic oedema and leukocyte infiltration significantly (Fig. 2a–c). DMTS therapy had no effect on pancreatic water content nor oedema during AP (Fig. 2b), but it did significantly reduce the leukocyte infiltration and MPO activity during the inflammation at both doses (Fig. 2c–d). Cellular damage was detected in the EtOH-POA AP model and reached ~ 35% (Fig. 2e). DMTS treatments with 3 × 75 and 3 × 100 mg/kg doses greatly reduced cellular damage. Interestingly, 3 × 75 mg/kg DMTS treatment was unable to reduce the severity of AP, when it was administered at 0, 3, and 12 h (Fig. S4).

**Figure 3:**
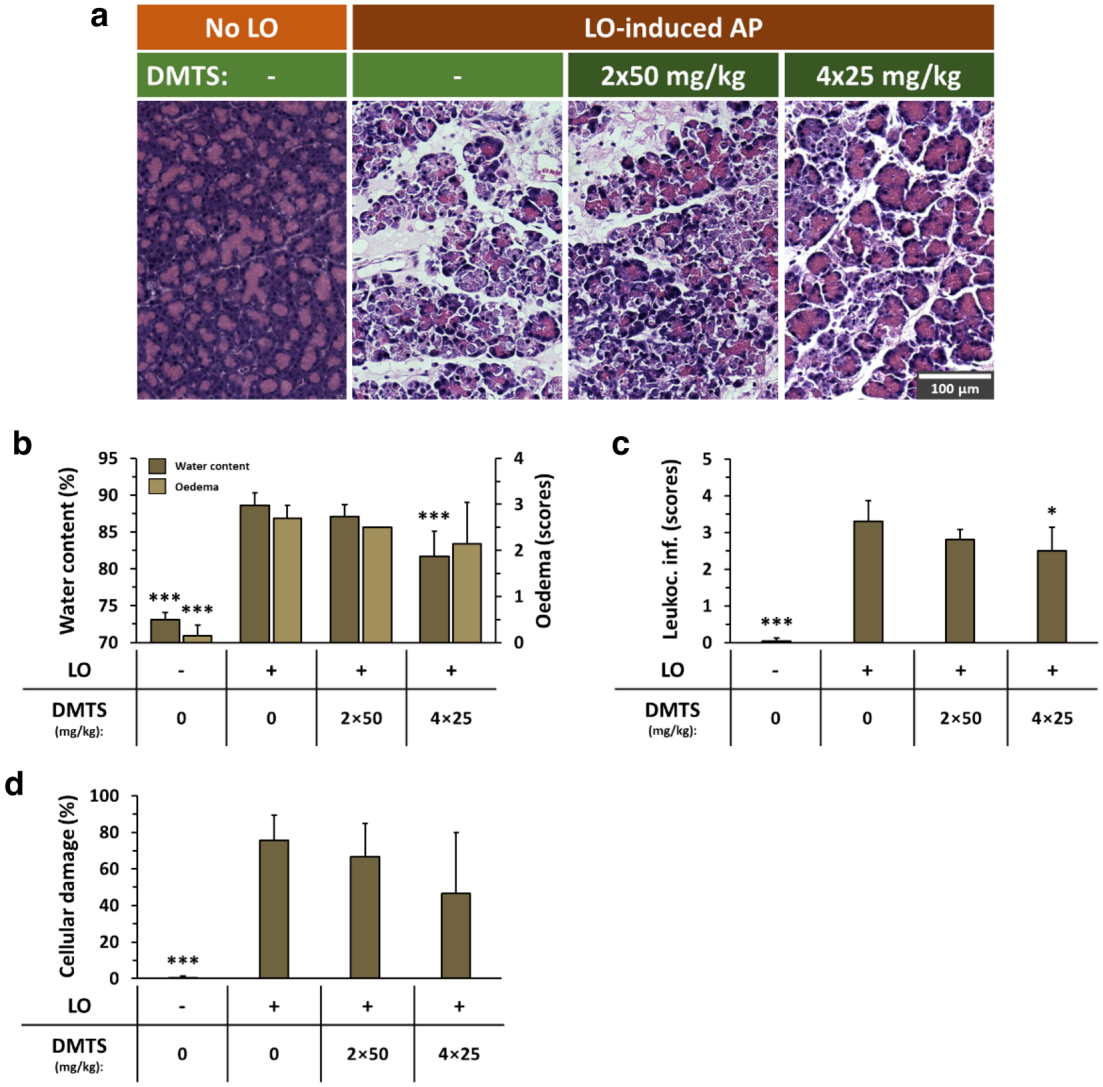
Dimethyl trisulfide (DMTS) treatment lowers the severity of L-ornithine-HCl (LO)-induced necrotising acute pancreatitis (AP) in rats. Rats were treated with 2 × 50 or 4 × 25 mg/kg DMTS subcutaneously, whereas intraperitoneal injection with 3 g/kg LO was employed to induce AP. Control animals received physiological saline rather than LO, or vehicle instead of DMTS. Animals were sacrificed at 24 h after the first LO or physiological saline injection. (**a**) Representative histopathological images of pancreatic tissues of the treatment groups. Bar charts display the extent of pancreatic (**b**) water content (as measured by the dry–wet weight ratio) and oedema (evaluation of histological sections), (**c**) leukocyte infiltration, and (**d**) cellular damage. Values represent means with standard deviation (SD). The total number of animals was 27, for details of exact means, SDs, and animals per group please view Supplementary Table 1. (**c**–**d**) One-way ANOVA was performed followed by Dunnett’s post-hoc test where all of the groups were compared to LO only group, *p < 0.05; ***p < 0.001. (**b**) Kruskal–Wallis test was performed followed by Dunn’s post-hoc test, the groups were compared to the LO only group, ***p < 0.001.

L-ornithine-HCl (LO) induced necrotizing AP in Wistar rats was also investigated to exclude any possible species-specific effects (Fig. 3). The LO-induced AP increased pancreatic water content, leukocyte infiltration, and tissue injury. DMTS doses of 2 × 50 and 4 × 25 mg/kg were used without AP and had no effect on the pancreas compared to the control (Fig. S5). Only the 4 × 25 mg/kg DMTS dose reduced the pancreatic water content and the amount of leukocyte infiltration during AP (Fig. 3b–c). DMTS administration did not significantly affect the very severe tissue damage (~ 75%) caused by the disease, only a tendency for DMTS protection was observed when AP was compared with AP + 4 × 25 mg/kg DMTS group (p = 0.064; Fig. 3d).

### Pain sensation and behavioural test

**Figure 4:**
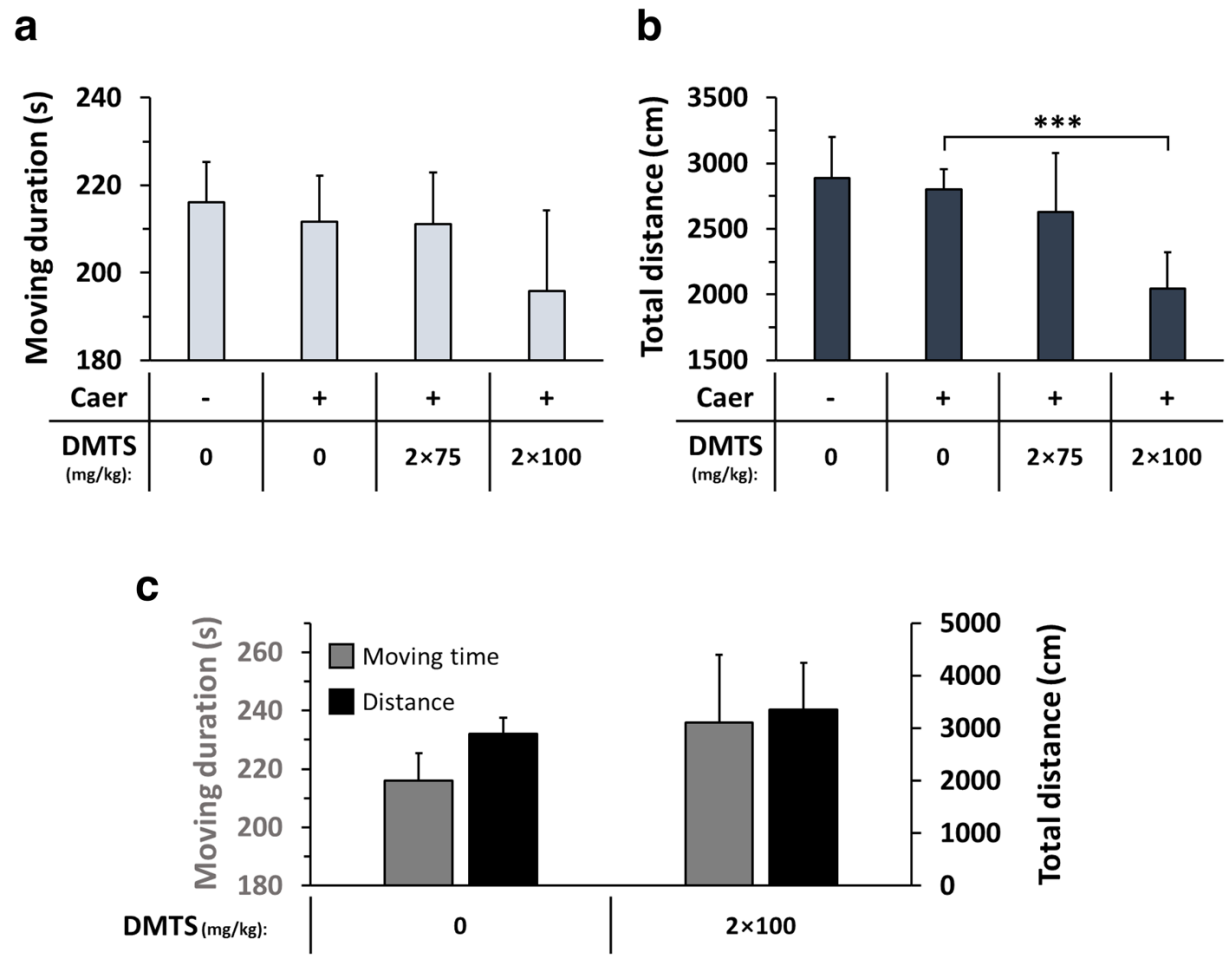
High-dose dimethyl trisulfide (DMTS) treatment lowers spontaneous motor activity in caerulein (Caer)-induced acute pancreatitis (AP) in mice. Open field test was used for the measurements, where the animals were placed into a 60 × 60 × 60 cm open area. (**a**) Moving duration and (**b**) total distance covered by animals with Caer-induced AP after various doses of DMTS treatment (2 × 75 mg/kg and 2 × 100 mg/kg subcutaneously (s.c.)). The larger dose of DMTS (2 × 100 mg/kg s.c.) had no effect on motor activity in mice without pancreatitis (**c**). Values represent means with standard deviation (SD). The total number of animals was 39, for details of exact means, SDs, and animals per group please view Supplementary Table 1. One-way ANOVA was performed in the case of (**a**) and (**b**) followed by Dunnett’s post-hoc test, and in panel (**c**) Student’s t-test was done. Statistically significant difference was marked with *** *p* < 0.001.

Because pain is one of the most important symptoms of AP, nociception was assessed in FVB/N mice using the von Frey test. Interestingly, no significant difference in abdominal pain was detected between control and Caer-induced AP groups, therefore the experiments were terminated (unpublished results). Since animal behaviour might appropriately reflect the extent of pain, open field observations were also conducted (Fig. 4). Although AP had no effect on movement duration and total distance covered by the FVB/N mice (Fig. 4a,b), the highest dose of DMTS (2 × 100 mg/kg) in AP reduced total distance compared to the AP group without DMTS treatment (Fig. 4b). The 2 × 100 mg/kg DMTS dose affected neither movement time nor total distance covered compared to the control in mice with no AP (Fig. 3c).

### DMTS induces HSP72 expression during acute pancreatitis

**Figure 5:**
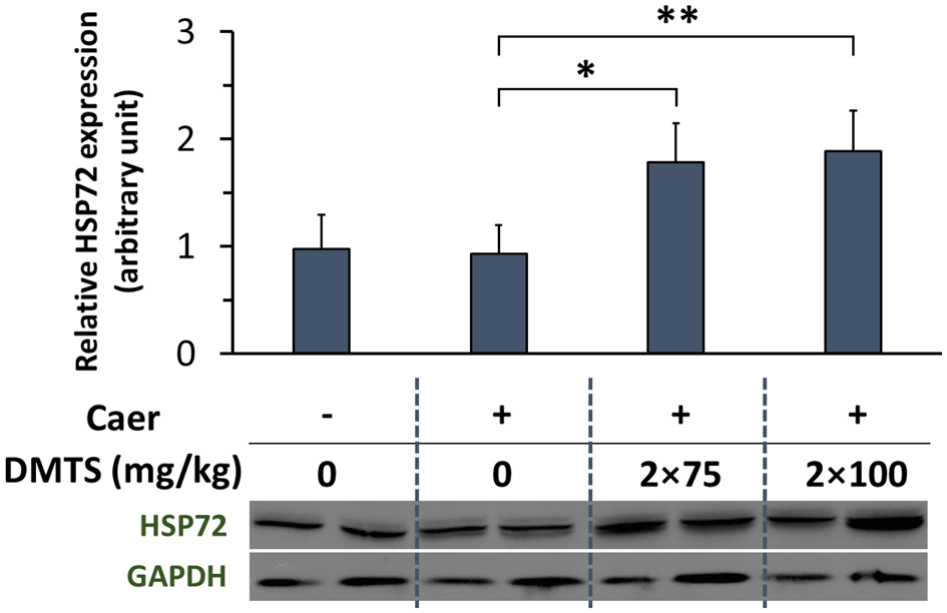
Alterations in heat shock protein 72 (HSP72) levels in acute pancreatitis (AP) in mice treated with dimethyl trisulfide (DMTS). Representative Western blot image of pancreatic HSP72 expression is depicted in the bottom, and the bar chart shows the densitometry of Western Blot images for pancreatic HSP72 level. Band intensities were assessed by using ImageJ software and HSP72 expression was normalized to glyceraldehyde-3-phosphate dehydrogenase (GAPDH) levels. Fig. S12 presents full blot images. Values represent means with standard deviation (SD). The total number of animals was 16, for details of exact means, SDs, and animals per group please view Supplementary Table 1. One-way ANOVA was performed followed by Dunnett’s post-hoc test. Statistically significant differences were marked with: *p < 0.05; **p < 0.01. Abbreviations: Caer, caerulein.

In mammalian cells, HSP72 is a stress-induced protective chaperone of the HSP70 family. HSP72 expression was not increased by DMTS alone (Fig. S6) nor was it elevated by Caer-induced AP after 12 h (Fig. 5). However, both doses (2 × 75 and 2 × 100 mg/kg) of DMTS significantly increased the HSP72 level compared to the AP group.

### DMTS has cytoprotective effects

**Figure 6:**
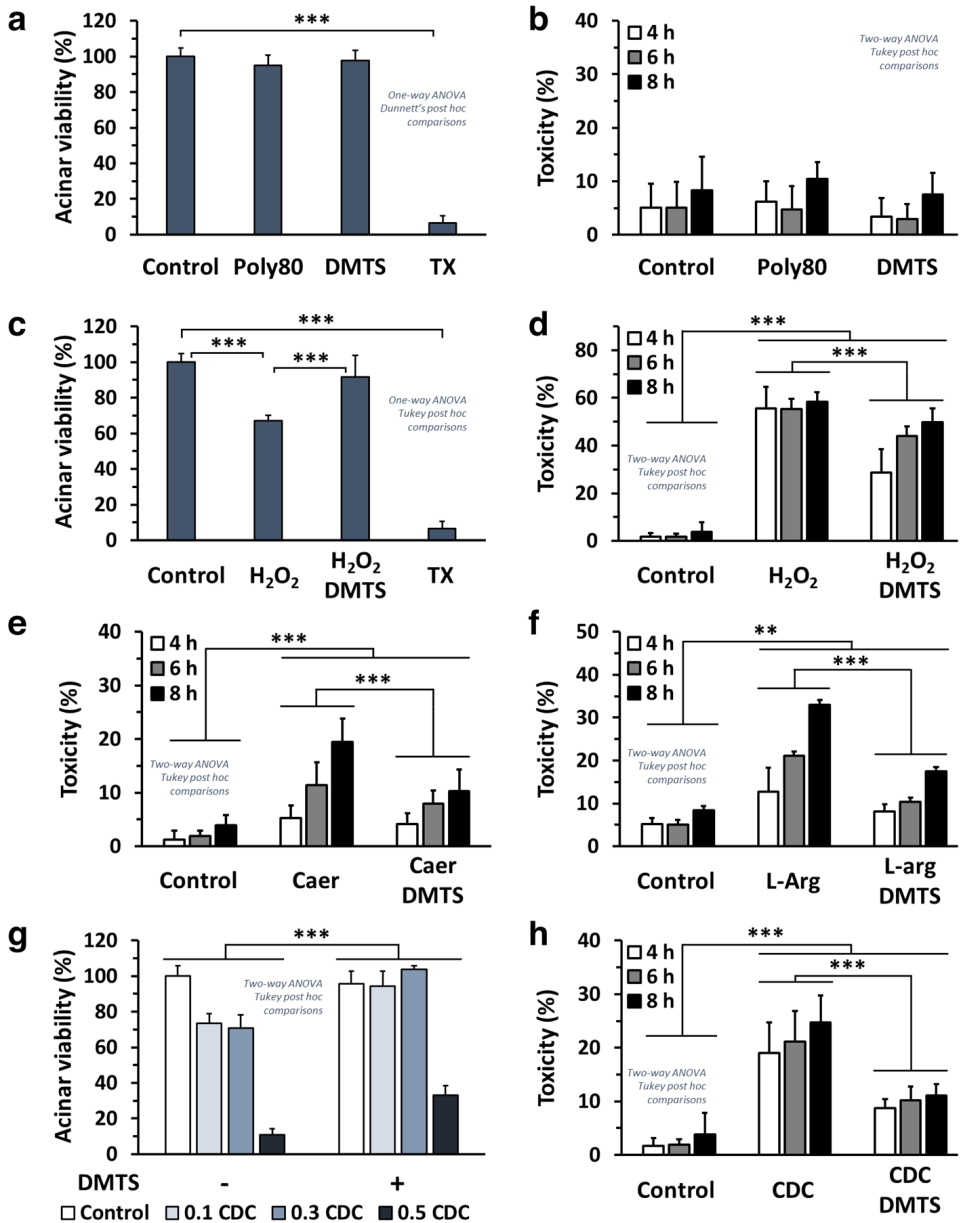
Dimethyl trisulfide (DMTS) exerts cytoprotective effects against reactive oxygen species (ROS) and acute pancreatitis (AP) inducing agents in isolated mouse pancreatic acinar cells. 3-(4,5-dimethylthiazol-2-yl)-2,5-diphenyltetrazolium bromide (MTT) method, indicated as acinar viability, was performed after 8 h treatment; and the propidium iodide (PI) method, indicated as toxicity, was used after 4, 6, and 8 h treatments. (**a**, **b**) The effect of 30 µg/ml DMTS and its vehicle (90 µg/ml Polysorbate 80) on acinar cells was determined. The effect of 500 µM H_2_O_2_ and its combination with 30 µg/ml DMTS on acinar cells was measured by (**c**) MTT and (**d**) PI methods. Investigation of how (**e**) 1 nM caerulein (Caer), or (**f**) 60 mM L-arginine-HCl (L-arg) and their combination with 30 µg/ml DMTS cause cellular toxicity was measured by the PI method. Triton X-100 (TX) was used as a positive control causing 100% toxicity. (**g**) The effect of sodium chenodeoxycholate (CDC; 0, 0.1, 0.3, 0.5 mM) and its combination with 30 µg/ml DMTS on acinar cells was measured by the MTT method at 8 h. (**h**) The PI method was applied after 4, 6, and 8 h to test the effect of 0.5 mM CDC and/or 30 µg/ml DMTS on acinar cells. Values represent means with standard deviation (SD). For MTT measurements 4–6 wells per group were used (a total of 68 wells), and the cells were derived from 2 animals. For the PI method 6 parallels were used (a total of 270 wells), and the cells were derived from 3 animals. For details of exact means, SDs, and animals per group please view Supplementary Table 1. One- or two-way ANOVA was performed followed by Dunnett’s or Tukey’s post-hoc tests (indicated in each plot). Statistically significant differences were marked with: **p < 0.01; *** *p* < 0.001.

Based on the pharmacokinetics of DMTS in mice^24^, its plasma concentration can reach 25–30 µg/ml. Therefore, the 30 µg/ml DMTS concentration was chosen for the in vitro experiments. Initially, the effect of DMTS (30 µg/ml) and its vehicle, 90 µg/ml Polysorbate 80 (Poly80), was tested on mouse pancreatic acinar cell viability by the 3-(4,5-dimethylthiazol-2-yl)-2,5-diphenyltetrazolium bromide (MTT) and propidium iodide (PI) methods (Fig. 6a,b). DMTS or its vehicle did not affect cellular viability and was not cytotoxic within 8 h. Treatment with 500 µM hydrogen peroxide (H_2_O_2_) significantly reduced acinar viability (Fig. 6c) and evoked toxic effect (Fig. 6d). In contrast, 30 µg/ml DMTS effectively restored the viability and reduced the H_2_O_2_-induced toxicity. AP inducing agents, namely Caer, L-arginine-HCl (L-Arg), and sodium chenodeoxycholate (CDC) were investigated on acinar cells with or without DMTS. Caer 1 nM (Fig. 6e) and 60 mM L-Arg (Fig. 6f) treatments resulted in 5–19% and 19–33% toxicity between 4 and 8 h treatments, but 30 µg/ml DMTS significantly reduced their adverse effects, measured by the PI method. All applied CDC concentrations (0.1, 0.3, and 0.5 mM) reduced the viability of acinar cells from 100% to 73 ± 6.8, 71 ± 7.4, and 11 ± 3.2% respectively in the MTT test (Fig. 6g). The 0.5 mM concentration of CDC caused 19 ± 5.6, 21 ± 5.7, and 25 ± 5.0% toxicity at 4, 6, and 8 h, respectively (Fig. 6h). 30 µg/ml DMTS significantly reduced the cytotoxic effect of CDC and increased viability of the cells, indicating its cytoprotective effects (Fig. 6g,h).

### DMTS modulates physiological, but not pathophysiological intracellular Ca^2+^ signalling in acinar cells

**Figure 7:**
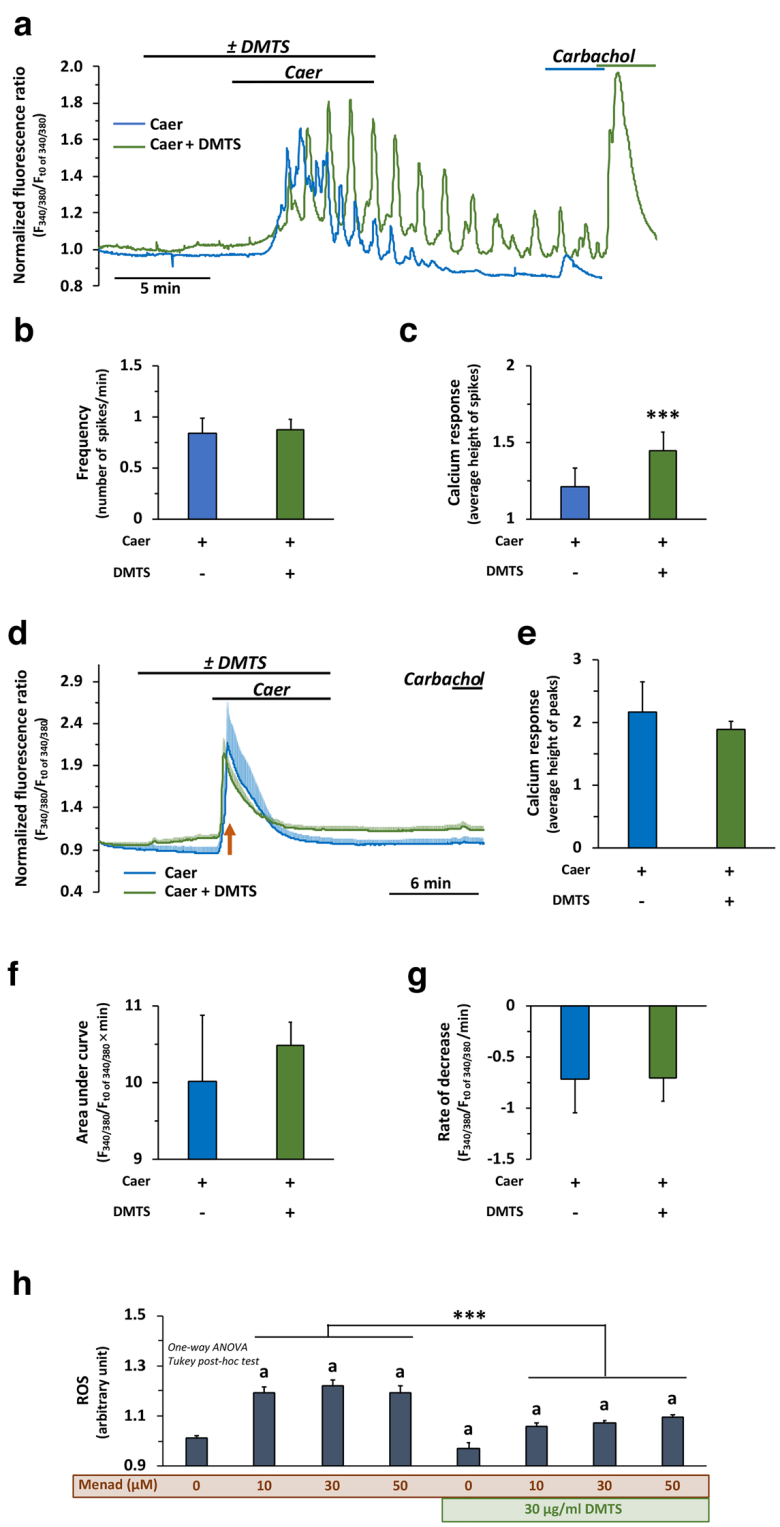
The effect of dimethyl trisulfide (DMTS) on intracellular Ca^2+^ signalling and its antioxidant property in mouse pancreatic acinar cells. (**a**) Representative trace of intracellular Ca^2+^ concentration (ic[Ca^2+^]) in response to treatment with 0.1 nM caerulein (Caer) with or without 30 µg/ml DMTS. At the end of the observation, acinar cells were subjected to 100 µM carbachol. The number (shown as frequency) and height (shown as calcium response) of individual spikes recorded between 480 and 960 s (8 min) were counted and plotted on panels **b** and **c**, respectively. (**d**-**g**) Treatment of cells with 1 nM Caer with or without 30 µg/ml DMTS. At the end of the experiment, cells were subjected to 100 µM carbachol. When the ic[Ca^2+^] reached the maximum (8 ± 0.5 min) the values were plotted on panel (**e**). The area under the curve was determined in case of 1 nM Caer treatment (**f**), and the slope (decreasing part) of the response on Caer treatment (**g**). (**h**) Menadione (Menad) treatment at 10, 30, and 50 µM with or without 30 µg/ml DMTS. Values represent means with standard deviation (SD). For the reactive oxygen species (ROS) method 5 parallels were used (a total of 40 wells), and the cells were derived from 2 different animals. In the case of ic[Ca^2+^] a total of 20 measurements were performed, and the cells were derived from 4 different animals. For details of exact means, SDs, and animals per group please view Supplementary Table 1. Statistics: (**h**) One-way ANOVA was performed followed by Tukey’s post-hoc test; and (**b**, **c**, **e**–**g**) Student’s t-tests were applied. Statistically significant differences were marked in the following manner: *** *p* < 0.001; ‘a’, vs. control (*p* < 0.05); ‘b’, vs. H_2_O_2_ (*p* < 0.05).

Intracellular Ca^2+^ concentration (ic[Ca^2+^]) was monitored in real-time in isolated pancreatic acinar cells. DMTS alone did not affect ic[Ca^2+^] (Fig. S7). To investigate the effect of Caer on ic[Ca^2+^], cells were perfused with maximal (0.1 nM) and supramaximal (1 nM) concentrations of Caer (Fig. 7a–e). Application of 0.1 nM Caer resulted in global, oscillatory increases in ic[Ca^2+^] (Fig. 7a). The Ca^2+^ stores were depleted thereafter and some minutes later carbachol, an acetylcholine (ACh) receptor agonist, was unable to elicit a relevant Ca^2+^ signal. DMTS pre-treatment had no effect on the Caer-evoked oscillation frequency (Fig. 7a,b), but there was a significant increase in the average height of Ca^2+^ signals when Caer treatment was compared to Caer + DMTS treament (Fig. 7a,c). Additionally, in the Caer + DMTS group, a remarkable increase in ic[Ca^2+^] could be registered in response to carbachol at the end of the experiment. The supramaximal concentration of Caer (1 nM) evoked an initial peak in the Ca^2+^ signal followed by a return to the basal levels and showed no further increase or oscillation during stimulation (Fig. 7d–g). Pre-treatment with DMTS did not affect the Caer-induced pathological Ca^2+^ signals.

### Exocrine and endocrine pancreatic cells express the *Trpa1* mRNA

Previous research has demonstrated that DMTS acts as a TRPA1 agonist^21^, and this ion channel activity can influence the course of AP^25^. Sensory nerves and stellate cells in the pancreas have been demonstrated to express TRPA1 channel^26^; however, it is unknown whether pancreatic acinar, ductal, or endocrine cells have this protein in their membranes, which may be modulated by DMTS directly. That is why we wanted to reveal whether *Trpa1* mRNA was expressed in different pancreatic cells (Fig. S8). RNAscope in situ hybridization technique was used to visualize *Trpa1* and *Urocortin3* (Langerhans islet marker) mRNAs in pancreatic tissue and this method was combined with immunofluorescence staining to amylase (acinar marker) co-staining. *Trpa1* mRNA expression was found in pancreatic acini that were stained for amylase (Fig. S8A–B). Pancreatic ducts were indirectly observed based on nucleus shape, luminar structure, and amylase positivity in the lumen (Fig. S8C–D). *Trpa1* mRNA expression was detected in ductal cells as well. Furthermore, *Urocortin3* positive endocrine cells also showed positive *Trpa1* mRNA expression (Fig. S8E–F). Based on these findings, DMTS could affect cellular signalling through *Trpa1*, but further studies are needed to reveal the background mechanisms.

### DMTS reduces oxidative stress in mouse pancreatic acinar cells

To investigate the antioxidant properties of DMTS, the general oxidative stress indicator 6-carboxy-2',7'-dichlorodihydrofluorescein diacetate (carboxy-H_2_DCFDA) was used in mouse pancreatic acinar cells keeping in mind all the limitations that are associated with these measurements^27^ (Fig. 7h). DMTS alone in the 1–60 µg/ml concentration range did not induce biologically relevant ROS production (Fig. S9). Via a redox cycling mechanism, menadione promotes intracellular ROS production^28^. All applied menadione concentrations (10, 30, 50 µM) increased acinar ROS levels (Fig. 7h), but the 30 µg/ml DMTS significantly reduced the effect of menadione on ROS formation.

### DMTS increases serum sulfide and persulfide levels

**Figure 8:**
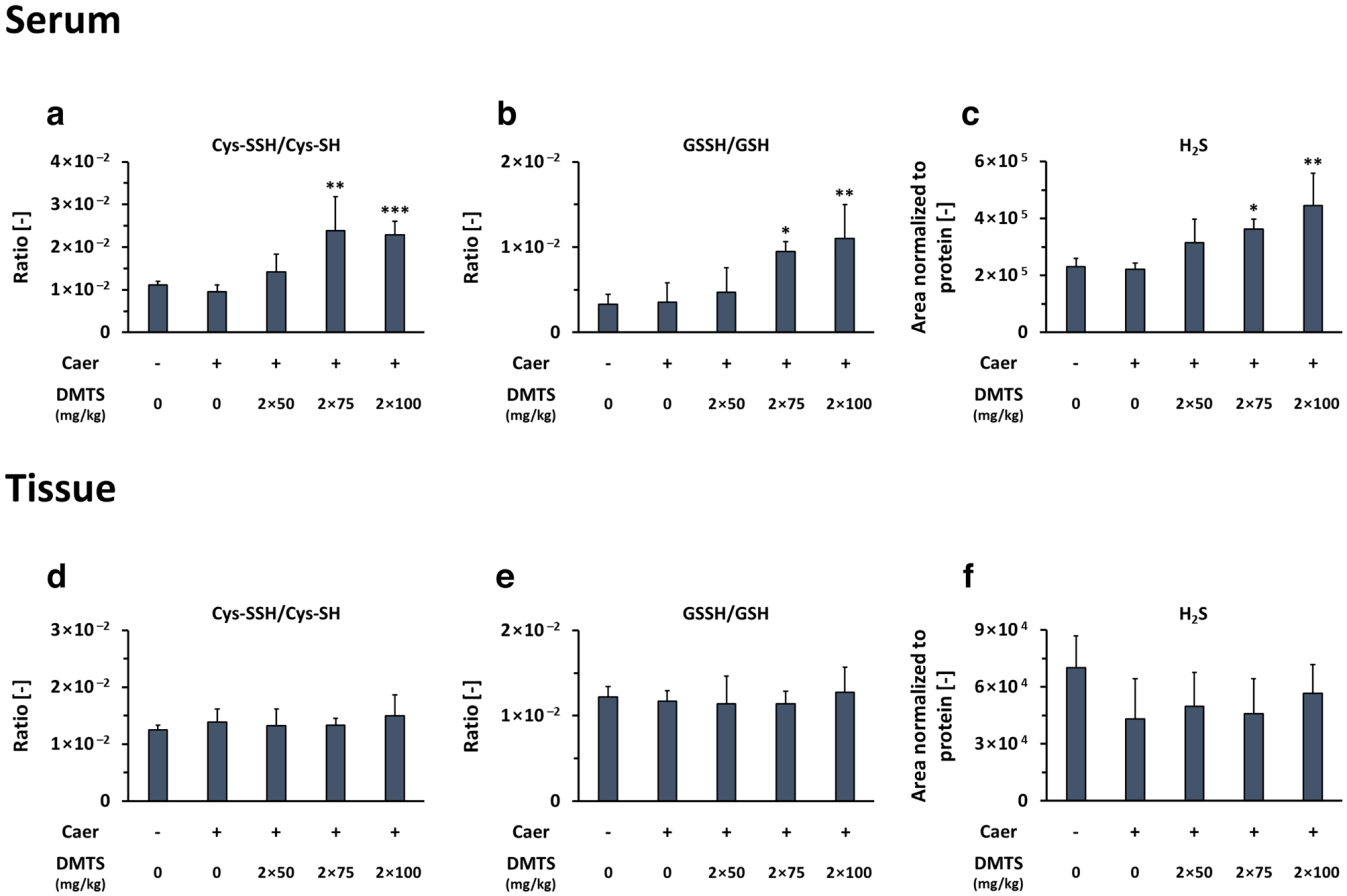
Dimethyl trisulfide (DMTS) elevates serum sulfide and persulfide levels but does not affect pancreatic persulfidation and sulfide. The bar charts show cysteine (Cys-SH) and glutathione (GSH) persulfidation levels in serum (**a, b**) and pancreatic tissue samples (**d**, **e**), respectively. Sulfur metabolome analyses of sulfide (H_2_S) levels in serum (**c**) and tissue samples (**f**). Values represent means with standard deviation (SD). The total number of animals was 22, for details of exact means, SDs and animals per group please view Supplementary Table 1. (**b**, **d**–**f**) One-way ANOVA was performed followed by Dunnett’s post-hoc test where all of the groups were compared to the Caer-only group, *p < 0.05; **p < 0.01. (**a**, **c**) Kruskal–Wallis test was performed followed by Dunn’s post-hoc test, the groups were compared to the Caer-only group, *p < 0.05; **p < 0.01; ***p < 0.001. Cys-SSH, cysteine persulfide; GSSH, glutathione persulfide.

To gain further insights into the protecting effects of DMTS against AP, HPLC–MS/MS (high performance liquid chromatography coupled with tandem mass spectrometry) based sulfur metabolome analyses were carried out. Mouse pancreatic tissue and serum samples were derived from Caer-AP model (12 h sacrifice time) treated with 2 × 75 and 2 × 100 mg/kg DMTS doses. In serum, we observed no significant changes in steady-state cysteine (Cys-SH) levels compared to the Caer-only group (Fig. S10A). However, we found that both 2 × 75 and 2 × 100 mg/kg DMTS resulted in markedly elevated levels of serum cysteine persulfidation (Cys-SSH) and consequently increased the persulfide ratio (Cys-SSH/Cys-SH) in AP compared to the Caer-only treatment (Fig. S10B and 8A). The level of glutathione (GSH) was diminished by Caer in the serum, which was reversed by all DMTS doses (Fig. S10C). Moreover, both 2 × 75 and 2 × 100 mg/kg DMTS significantly increased the levels of glutathione persulfide (GSSH) and the GSSH/GSH ratio (Fig. S10D and 8B). We also observed an elevated level of sulfide (H_2_S) in the serum resulting from both DMTS doses in AP compared to the Caer-only group (Fig. 8c).

In the pancreatic tissue, the analyses revealed no significant difference in any of the measured metabolite levels between Caer groups with or without DMTS treatment (Fig. 8d–f and S9I–L). Lanthionine (Lanth), homolanthionine (HLanth) and cystathionine (CTH) are indicative by-products of H_2_S generation by cystathionine β-synthase (CBS) and CSE enzymes. These metabolite levels remained unchanged among control and treatment groups both in serum and tissue samples (Fig. S10E–F and S10M–P), and this indirectly suggests that the classical CBS and CSE sulfide-producing pathways were not altered. Overall, our results showed that DMTS elevates the serum level of sulfide as well as low molecular weight persulfides and reverts the effect of Caer on GSH, but it did not affect the levels of any of the measured metabolites in pancreatic tissue.

## Discussion

Organosulfur molecules have huge potential as drug candidates in different diseases, particularly in conditions associated with inflammation. In this research, we found that the organosulfur family member DMTS could effectively reduce the severity of AP in three different models of the disease. Likewise, DMTS exerted cytoprotection against several AP inducing agents as well as against cellular oxidative stress, and we demonstrated that DMTS is a sulfur donor molecule.

DMTS was tested in several doses in mice using Caer- and EtOH-POA-induced necrotizing AP models, and in rats using LO to induce AP. In mice, DMTS dose-dependently reduced the severity of Caer-AP. DMTS administration lowered pancreatic leukocyte infiltration, MPO activity, and cellular damage, but it had no effect on the tissue oedema. TNF-α or IL-1β are important markers of pancreatic inflammation in AP, however, they are expressed in the early phase of Caer-induced AP (6–10 h; please see Fig. S11). Therefore, these cytokines were not investigated in the organosulfur experiments due to the late timepoints of animal sacrifice. In the EtOH-POA AP model, DMTS significantly reduced the leukocyte infiltration and the cellular damage, when it was applied 3–12 h later following the initiation of AP. Interestingly, the treatment arrangement remarkably affected the outcomes. Too early administration (0, 3, and 12 h) of the same doses of DMTS did not affect the disease severity. Most probably the short half-life of DMTS (36 min) could be the major cause of this, the serum concentration of DMTS is remarkably lowered by that time when the inflammation can be decreased^29^. In rats with LO-induced AP, DMTS only reduced the leukocyte infiltration and had negligible effect on the cellular damage in the pancreas. Diallyl disulfide, a similar molecule to DMTS, was also shown to exert a protective effect on Caer-induced AP in mice in another study^17^. It reduced the pancreatic tissue injury and the damage in the lungs resulting from AP. Diallyl disulfide decreased TNF-α, CSE, neurokinin-1 receptor and preprotachykinin A expression in pancreas and lung, and the production of H_2_S. Diallyl disulfide also decreased the serum amylase activity in AP mice. DMTS treatment facilitates methaemoglobin formation which results in darker blood colour^20^. Most likely this darker colour has interfered with our serum amylase activity measurements. Therefore, we could not determine any differences in serum amylase activity. Other organosulfur molecules and H_2_S donors, like S-diclofenac, S-propargyl-cysteine, and the naturally occurring sulforaphane, also reduced the severity of AP and/or the concomitant lung injury^18,19,30^. In general, these previous findings and our in vivo studies indicate a potential of organosulfur compounds as drug candidates in AP.

Organosulfur molecules can influence leukocyte infiltration and their products during inflammation. GYY4137 a slow-releasing H_2_S donor, reduced the cytokine production of murine macrophages^31^. Pálinkás et al. demonstrated that H_2_S interacts with and inhibits the MPO generated by neutrophils^32^. Sulforaphane also reduced MPO production^30^. H_2_S inhalation decreased neutrophil transmigration into the lung^33^. Similar to these results, we also observed, that DMTS administration reduces the number of pancreatic leukocytes and the total tissue MPO activity.

HSPs with chaperon properties (HSP27, HSP60, HSP72) can be upregulated during experimental AP^34–36^ and exert protection. It was shown that exogenous H_2_S from sulfide donors stimulates elevation of HSP72 which may contribute to decreased myocardial injury during ischaemia-reperfusion^37^. We observed that treatments with DMTS in AP enhanced the HSP72 expression compared to the AP group without DMTS administration. This rise in chaperone protein levels could contribute to the protective effect of DMTS. Interestingly, only DMTS treatment in physiological conditions did not alter the HSP72 level, thus the DMTS is an HSP72 coinducer (induction of HSPs during the course of the disease). Similar results were seen earlier with BRX-220, which is also an HSP protein coinducer^38^. It should be noted that increased HSP72 expression could also indicate toxicity. Since DMTS alone did not affect HSP72 levels, it may indicate that DMTS alone is not toxic to pancreatic tissue.

One of the most important symptoms and diagnostic criteria for AP is intensive abdominal pain, which is present in more than 95% of the patients^3,39^. Generally, pain is associated with anxiety-like behaviour, and open field tests could reveal this association^40,41^. As DMTS has analgesic effects^21^, the nociception and the behaviour of the animals were examined in AP following DMTS treatment. Unfortunately, the von Frey test, which was used for detecting nociception, could not reveal any differences between the control and AP groups. Similar outcomes were observed earlier by Durst et al. in C57BL/6 mice^42^. Nevertheless, Michalski et al. could detect allodynia and hyperalgesia with the von Frey test in AP^43^. These contradictory results recommend carefully interpreting the data and finding other solutions to detect nociception in AP. In accord with our findings, the behaviour of mice did not change in AP groups compared to the control in the open field test^43^. Interestingly, the highest dose of DMTS reduced the total moving distance during AP. Similar results were observed with H_2_S donor molecules, like NaHS or organic disulfides, they reduced the movement of mice^44,45^. The exact cause of this phenomenon is unknown, but cyanosis through converting haemoglobin to methaemoglobin^20^ in response to DMTS treatment could contribute to this effect^19^. Nevertheless, this requires further investigation.

DMTS alone or the vehicle did not show any pancreatic acinar cell toxicity, nor did it alter the metabolic activity of the cells. Contrarily, H_2_O_2_ and AP-inducing agents (Caer, L-Arg, and CDC) led to cellular injury. All these cytotoxic effects were significantly reduced by DMTS administration. Several organosulfurs have in vitro cytoprotective effects, including diallyl trisulfide, GYY4137, ATB-346, or cysteine persulfides^46–48^. These effects can be associated with several different processes. These include protection of protein function through protein persulfidation^49,50^, activation/inactivation of certain signalling pathways to counteract the effects of oxidative stress or the activation of Kelch-like ECH associated protein 1/Nrf2 system^47–49,51,52^.

Intracellular Ca^2+^ signalling is an important process regulating cellular activities. However, pathophysiological events can cause abnormally elevated ic[Ca^2+^], which in the case of acinar cells will initiate AP^53,54^. High but physiological concentration of Caer (0.1 nM) triggered global ic[Ca^2+^] oscillations, but these oscillations diminished within 5–7 min and then the ACh receptor agonist carbachol could not stimulate further Ca^2+^ release from intracellular stores. Nevertheless, when DMTS pre-treatment was applied, the 0.1 nM Caer-induced Ca^2+^ oscillations were sustained for longer periods and carbachol could evoke marked increase in ic[Ca^2+^]. We suppose that the intracellular Ca^2+^ stores become exhausted in response to maximal Caer (0.1 nM) stimulation and DMTS can prevent this. In addition, acinar cells become unresponsive to carbachol after maximal Caer administration, but their responsiveness was kept in the presence of DMTS. However, DMTS treatment did not affect the cellular ic[Ca^2+^] response when supramaximal and pathological concentrations of Caer (1 nM) was applied. Based on these observations, we can conclude that DMTS can moderate physiological, but not pathophysiological ic[Ca^2+^].

Sulfur donor molecules, like polysulfides and H_2_S are effective antioxidants^55^. This property can contribute to their beneficial effect on various diseases. During AP, in response to increased ic[Ca^2+^], a significant amount of ROS (e.g. H_2_O_2_) is generated by acinar and inflammatory cells^56–58^. Among different processes, the oxidative stress and mitochondrial Ca^2+^ influx causes the opening of mitochondrial transitional pores in acini, which will lead to reduced mitochondrial membrane potential (ΔΨ_*m*_). This reduced ΔΨ_*m*_ inhibits the production of ATP in acinar cells and further exacerbates the sustained and global increase in ic[Ca^2+^]^59^. Additionally, excessive ROS generation and ATP depletion promote necrotic cell death rather than apoptosis^56^, and the resulting necrosis is also among the hallmarks of the serious form of the disease. Thus, we tested how DMTS protects primary acinar cells during oxidative stress. DMTS was protective when acinar cells were treated with oxidative stress inducer, menadione, confirming its antioxidant capability. We hypothesised what are the background mechanisms of this antioxidant effect. DMTS contains a sulfane sulfur (S^0^), which makes it redox reactive^60^. Via transpersulfidation, DMTS can act as a sulfane sulfur donor generating low molecular weight persulfides or persulfidate protein cysteine residues to conserve their activity by utilizing the thioredoxin system^49^. Beyond its direct effect, DMTS as a similar molecule to diallyl trisulfide, may also induce antioxidant enzymes through the Nrf2 pathway in the later phase, but it needs additional investigation^61,62^. As demonstrated above, excessive ROS production leads to ATP depletion and necrosis. It would also be interesting in the future to test intracellular ATP production during AP and DMTS treatment. Petersen et al. described further mechanisms related to oxidative stress and necrosis^59^. Acinar necrosis in response to sustained ic[Ca^2+^], ΔΨ_*m*_ loss, and oxidative stress causes ATP, kallikrein, and trypsin release to the extracellular space. ATP and kallikrein will then activate macrophages and stellate cells, respectively, and cause ic[Ca^2+^] elevations^63^. Thus, further studies focusing on revealing how DMTS influences the disease pathophysiology should also investigate this aspect of the disease as well.

It has been proposed that H_2_S signalling occurs via oxidative posttranslational modification of cysteine residues to persulfides^64–66^. Via interaction with metalloproteins or protection of protein function from irreversible overoxidation, both H_2_S and protein persulfidation protect cells from oxidative stress^49,67^. Literature data showed that generally in inflammatory disease H_2_S production and increased persulfidation levels have protective roles, but their production are disrupted and decreased^12,68^. Interestingly, other studies focusing on AP described opposite effects, experimental AP caused an increase in H_2_S, with enhanced CBS and CSE expressions^15^. Our sulfur metabolome analysis showed that in experimental AP the serum and pancreatic tissue levels of sulfide and protein persulfidations were unchanged. Here, we should note that the detection methods for sulfide and persulfidation hugely impact the outcomes and differs between papers, and could explain the diverse results^69^. Therefore, validated detection methods for sulfide and persulfidation are needed, and the result should not be overinterpreted^70,71^. In our experiments, DMTS elevated the serum levels of sulfide and low molecular weight persulfides, while the sulfide-producing classical pathways, using CBS and CSE enzymes, were unchanged. This increase in the levels of persulfide can also indicate an increase in the generation of H_2_S. Our observations suggest that DMTS acts as an H_2_S/sulfane sulfur donor, which is clearly seen in the serum H_2_S levels and also manifested in increased protein persulfidation, which might contribute to the observed protective effects against oxidative or inflammatory processes. It is also noteworthy that even if H_2_S plays an important part in the possible mechanism of DMTS protecting against AP, at present it is very difficult to differentiate whether this effect is mediated by H_2_S or persulfides/polysulfides. This is due to the fact that the currently available state-of-the-art detection protocols are limited and may artificially alter the speciation of these reactive sulfur compounds^70,71^.

TRPA1 is a Ca^2+^ channel that acts as a sensor for pain, cold and itch. It is expressed in nerves, macrophages, pancreatic stellate cells etc.^21,23,26,72,73^. Our experiments have demonstrated that *Trpa1* mRNA is also expressed by pancreatic acinar, ductal and endocrine cells. Prior research showed that DMTS and polysulfides are TRPA1 agonist molecules and via this mechanism DMTS exerts analgesic and anti-inflammatory effects via the release of somatostatin^21,23,72,74^. Nevertheless, most types of TRPA1 agonists cause the release of calcitonin gene-related peptide (CGRP) and tachykinin from the sensory nerve endings, which lead to hyperalgesia and enhancement of inflammation. Other publications investigated the TRPA1 ion channel contribution to the acute inflammation of the pancreas^25,26^. Those publications showed that TRPA1 inhibition or gene deletion/silencing reduced the severity of experimental AP which was induced by Caer or EtOH-POA treatments. Based on all these observations, TRPA1 has mixed effect, and it can both increase or decrease the inflammation, depending on CGRP, tachykinin or somatostatin release, but the detailed background mechanisms should be revealed in the future. Furthermore, the interaction of DMTS with TRPA1 would be worth investigating later.

According to our results, we observed that DMTS exterts anti-inflammatory effects in experimental AP. In vitro we demonstrated that DMTS is cytoprotective, it modulates physiological Ca^2+^ signalling, reduces ROS levels, and it increases serum sulfide and protein persulfidation levels. These effects of DMTS could be caused by the upregulation of HSP72 expression, by its antioxidant properties, by being an H_2_S donor molecule, and/or by reducing leukocyte infiltration and inhibiting MPO activity.

## Methods

### Materials

All chemicals were purchased from Merck Life Science Kft. (Budapest, Hungary), unless indicated otherwise. Before each experiment, the solutions utilised for in vivo measurements were freshly prepared. DMTS stock solution of 10 mg/ml was prepared in 30 mg/ml Poly80 in physiological saline solution (PS) by stirring overnight. Stock solution of 4 mM Caer (Glentham Life Sciences, Corsham, UK) was prepared in dimethyl sulfoxide (DMSO) and the working solution of Caer (5 µg/ml) was diluted in PS. LO (300 mg/ml, pH = 7.4) was dissolved in PS.

### Animals

Eight- to ten-week-old male FVB/N mice weighing 22–30 g and four- to six-week-old Wistar rats (180–250 g) were used for the experiments. The animals were obtained from Charles River Laboratories Inc. (Wilmington, MA, USA). They were housed in the departmental animal facility at a constant room temperature of 23 °C with a 12-h light–dark cycle and were allowed free access to water and standard laboratory chow for rodents (Innovo Kft., Isaszeg, Hungary).

Our experiments were executed according to the European Union Directive 2010/63/EU and the Hungarian Government Decree 40/2013 (II.14.). Experiments involving animals were approved by both local (University of Szeged) and national ethical committees (X./1714/2020.; date: 24th July 2020). The study was also carried out in compliance with the ARRIVE guidelines^75^.

### In vivo experiments: acute pancreatitis induction, treatment with DMTS and tissue harvesting

**Figure 9:**
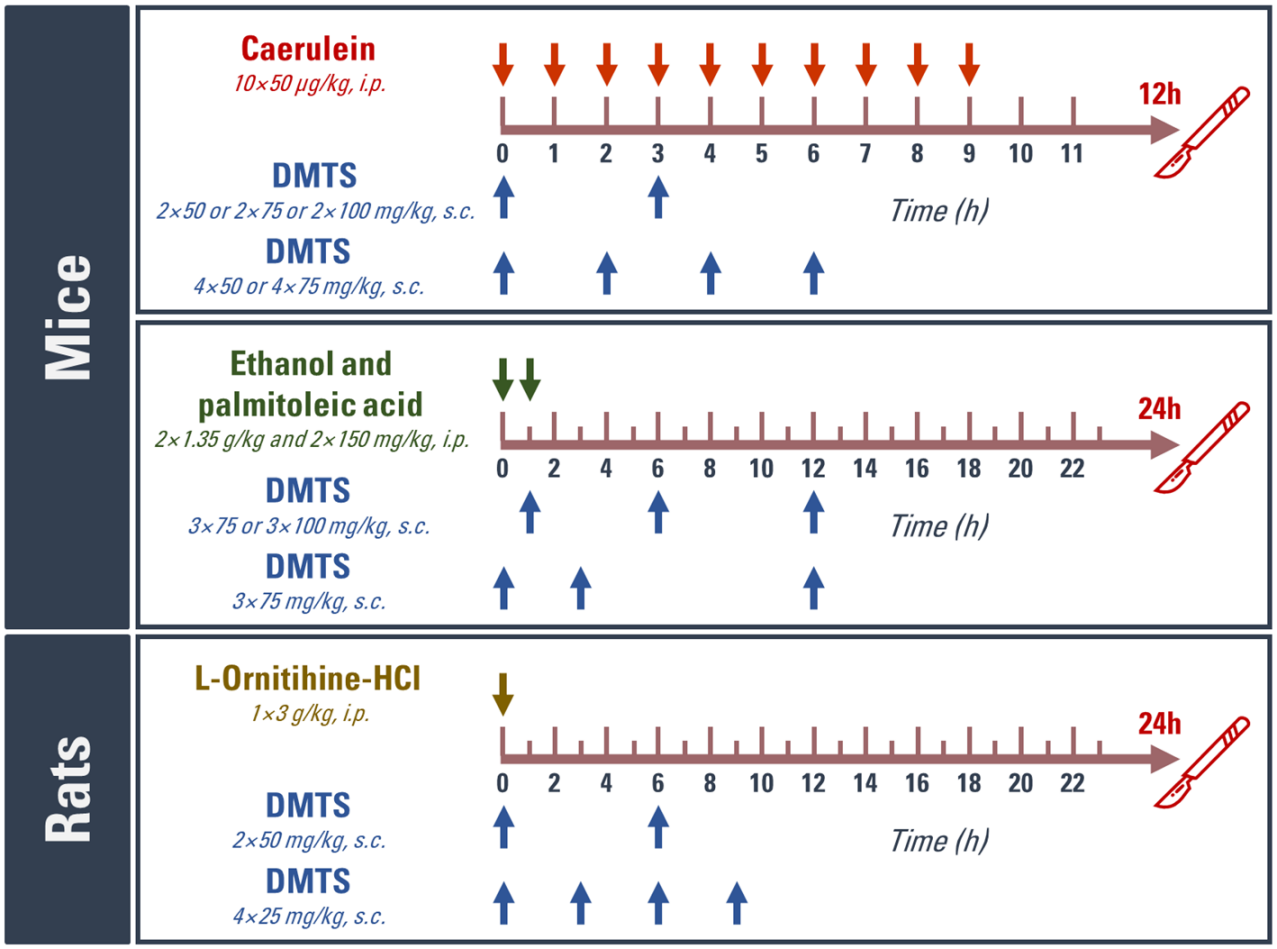
Schematic view of the in vivo experimental setup. Treatment arrangements for acute pancreatitis induction and administration of dimethyl trisulfide (DMTS) are depicted. Arrows above or below the timeline show the injections. Control animals received only vehicles. *i.p.* intraperitoneal; *s.c.* subcutaneous.

All in vivo experiments were performed in a dedicated laboratory space. The animals were randomly allocated into groups using a computer-based random order generator. No criteria for inclusion and exclusion were set. Three different necrotising AP models were employed and induced in the following manner: [Caer-AP model] intraperitoneal (i.p.) hourly injection of 10 × 50 µg/kg Caer in mice; [EtOH-POA model] hourly i.p. injection of 2 × 1.35 g/kg EtOH and 2 × 150 mg/kg POA in mice; [LO model] i.p. injection of 1 × 3 g/kg LO in rats (Fig. 9)^76–78^. DMTS treatments were applied s.c. to avoid interaction between DMTS and the AP-inducing agent. Mice with Caer-induced AP received 2 times 50, 75 or 100 mg/kg DMTS at 0 and 3 h, or they received 4 times 50 or 75 mg/kg DMTS at 0, 2, 4, and 6 h after the first Caer injection. Mice in EtOH-POA AP model received 3 times 75 or 100 mg/kg DMTS at 1, 6, 12 h; or 3 times 75 mg/kg DMTS at 0, 3, 12 h. Rats were injected with DMTS two times with 50 mg/kg dose at 0 and 6 h, or four times with 25 mg/kg dose at 0, 3, 6, and 9 h. Control groups were given PS solution instead of Caer, EtOH-POA, and Poly80 instead of DMTS.

Animals were sacrificed 12 h after the first Caer injection or 24 h after the first EtOH-POA or LO injections (at the peak of pancreatic inflammation). For this process, deep anaesthesia was induced with 45 mg/kg i.p. pentobarbital (Bimeda MTC, Cambridge, Canada). Blood was collected through cardiac puncture (~ 400 µl) and allowed to clot at room temperature for 30 min. Following centrifugation (3000 g at room temperature for 5 min), the serum was obtained and stored at –20 °C until use. The pancreas was quickly removed and was cleaned from fat and lymph nodes on ice, then it was cut into pieces. One large section was promptly frozen in liquid nitrogen and stored at –80 °C until biochemical assays were performed. Another piece of the pancreas was fixed in 8% neutral formaldehyde solution for histological examination. A third section was stored in Eppendorf tubes at room temperature for dry–wet weight measurement. To minimise subjective biases, various investigators were involved at different stages of each in vivo experiment (allocation and administering of the treatments, sacrification, outcome assessment, data analysis).

### Histological analysis

The formalin-fixed pancreatic samples were dehydrated and cleaned before being paraffin-embedded and sectioned to 3 µm. The sections after stained with hematoxylin and eosin were examined by two independent researchers blinded to the experimental protocol. Oedema was scored on a scale of 0 to 3 points (0: none; 1: patchy interlobular; 2: diffuse interlobular; 3: diffuse interlobular and intra-acinar), leukocyte infiltration on a scale of 0 to 4 points (0: none; 1: patchy interlobular; 2: mild diffuse interlobular; 3: moderate diffuse interlobular; 4: diffuse interlobular and intra-acinar). The percentage of cell damage was also evaluated, and all forms of cell death (like necrosis and apoptosis) were considered tissue damage.

### Laboratory measurements

The water content of pancreatic tissue was investigated. The wet weight (WW) of the pancreas was measured immediately after harvesting, then the tissues were dried for 48 h at 100 °C, and dry weight (DW) was measured as well. The wet/dry weight ratio was calculated as: [(WW–DW)/WW] × 100.

Pancreatic MPO activity, a hallmark of leukocytic infiltration, was determined from the excised pancreas samples. Pancreatic tissues (~ 30–50 mg) were thawed and chopped into small pieces before being homogenised in phosphate buffer (KH_2_PO_4_ 20 mM and K_2_HPO_4_ 20 mM buffer, pH = 7.4) using a Miccra D-9 Digitronic device (Art-moderne Laborteknik, Müllheim, Germany) on ice. Pellets were collected and resolved in a phosphate buffer (50 mM KH_2_PO_4_ and 50 mM K_2_HPO_4_ buffer, pH = 6.0) containing 0.5% hexadecyl trimethylammonium bromide detergent (1 ml buffer/sample) after centrifugation (5000 g at 4ºC for 20 min). Following thorough vortexing, homogenates were centrifuged at 5000 g at 4 °C for 20 min and aliquots of the supernatant were put in Eppendorf tubes. MPO activity was assayed using 3,3^′^5,5`-tetramethyl-benzidine-H_2_O_2_. Reactions were carried out in 96-well microplates at room temperature. The optical density (OD) at 620 nm was measured and plotted at 5 min intervals for 10 min, using a microplate reader (BMG Labtech, Ortenberg, Germany). The initial slope of the curve was used to calculate the reaction rate (ΔOD/time). A calibration curve was produced, with the rate of reaction plotted against the standard samples. Neutrophil granulocyte accumulation was assessed by comparing the MPO activity of the sample to the human myeloperoxidase activity of the standard preparation.

Pancreatic HSP72 expression was measured from tissue homogenate using Western blot analysis^79^. Sonication (Branson Sonifer 250; Emerson Electric, Brookfield, CT, USA) was used to homogenise pancreatic tissue on ice in a buffer containing: 10 mM Na-HEPES, 1 mM MgCl_2_, 10 mM KCl, 1 mM DL-dithiothreitol, 5 mM iodoacetamide, 4 mM benzamidine-HCl, 1 mM phenylmethyl sulfonylfluoride. The protein concentration of the homogenate was determined using the Bradford protein assay. 40 µg of protein were loaded per lane. The samples were separatedusing an 8% sodium dodecyl sulfate (SDS)-polyacrylamide gel. The gels were stained with Coomassie brilliant blue (to confirm equal loading of proteins for Western blot analysis) or transferred to a nitrocellulose membrane for 1 h at 100 V. The nitrocellulose membranes were blocked in 5% non-fat dry milk for 1 h before being incubated with rabbit anti-HSP72 (cat.no.: PA5-28,003; Thermofisher Scientific, Waltham, MA, USA) antibody for an additional 1 h at room temperature. GAPDH (cat.no.: PA1-987; Thermo Fisher Scientific, Waltham, MA, USA) was used as a loading control. The immunoreactive protein was visualized by enhanced chemiluminescence, using horseradish peroxidase-coupled anti-rabbit immunoglobulin at 1:5000 dilution for 1–5 min (Agilent Technologies, Santa Clara, CA, USA). Quantitative analysis of the results was achieved using the ImageJ software (NIH, Bethesda, MD, USA). The blot images were cropped, and only the relevant bands are shown in the figures. The original blots are presented in Fig. S12.

### Open field test

FVB/N mice were studied for changes in behaviour and motor activity. No pancreatitis was induced in the animals. DMTS treatments (2 × 75 mg/kg and 2 × 100 mg/kg s.c.) used in pancreatitis experiments were tested. Ten minutes after the first injection of DMTS or Poly80, mice were placed into the middle of a 60 × 60 × 60 cm open arena and were recorded for 7 min. The last five min were assessed with Noldus EthoVision XT 15 software. The videos were analysed in terms of time spent in active movement and the total distance travelled during this time.

### Pancreatic acinar cell isolation

Mouse pancreatic acinar cells were isolated with collagenase digestion according to Williams et al.^80^. After the animals were sacrificed, the pancreas was removed, washed with PS, and placed into 0 °C PS. The tissue was then cleaned from lymph nodes and fat. The Digest solution utilised in the next steps contained in DMEM/F12 the followings: 2 mM L-glutamine, 0.25 mg/ml soybean trypsin inhibitor (Thermo Fisher Scientific, Waltham, MA, USA), 2.5 mg/ml bovine serum albumin and 4500 U/g pancreas type 4 collagenase (Worthington Biochemical Co., Lakewood, USA). Following cleaning, the pancreas was cut into small pieces in 5 ml Digest solution. The tissue was incubated for 10 min in a shaking water bath (150 rpm) at 37 °C, then the digestion medium was changed with a fresh one and the tissue was incubated for another 40–50 min with shaking. After digestion, the acinar cells were washed twice by centrifugation at 4 °C, 50 rcf for 2 min, then resuspended in acinar cell culture medium which containing the followings in Medium 199: 2 mM L-glutamine, 0.25 mg/ml soybean trypsin inhibitor, 5% fetal bovine serum. The weight of the cellular supernatant was quantified by an analytical scale and the final cell suspension was set to 30 mg cells/ml. The cells were placed in a 37 °C CO_2_ incubator. The viability of acini was rapidly determined by the trypan blue technique, and they were used for in vitro measurements if their viability was > 90%.

### Acinar viability measurements

The MTT and PI methods were employed to determine cellular viability. The MTT reflects cellular metabolic activity, whereas PI indicates the cellular necrosis. In the case of the MTT assay, isolated pancreatic acinar cells (~ 15 mg cells/ml) were placed into a 96-well plate. Each of the following treatment solutions was prepared in acinar cell culture medium: 30 µg/ml DMTS; 90 mg/ml Poly80; 500 µM H_2_O_2_; sodium chenodeoxycholate at 0.1, 0.3, and 0.5 mg/ml concentrations; 10 mg/ml Triton X-100. Cells were treated with one of these treatment solutions or with a certain combination of them for 5 h at 37 °C. Then 0.5 mg/mL MTT dye (3-[4,5-dimethylthiazol-2-yl] -2,5 diphenyl tetrazolium bromide) was added to the cells, and they were incubated for additional 3 h with the dye and the treatments. After the 8 h treatment (5 and 3 h incubations) the formed formazan crystals were dissolved in DMSO. The absorbances were measured by FLUOstar OPTIMA (BMG Labtech, Ortenberg, Germany) plate reader by using a 595 nm filter. Triton X-100 treatment was the negative control, while the untreated cells were the positive controls (considered as 100% viability).

For the PI assay, acinar cells were seeded on 8-well microscope slides. The cells were treated with the following: 30 µg/ml DMTS; 90 mg/ml Poly80; 500 µM H_2_O_2_; 0.1 nM Caer; 60 mM L-Arg; sodium chenodeoxycholate at 0.1, 0.3, and 0.5 mg/ml concentrations; or 10 mg/ml Triton X-100. The cells were also administered 1 µg/ml PI and 1.87 µg/ml H33342, where PI stained the nuclei of membrane damaged or necrotic cells, while the H33342 stained all the cell nuclei. Following 4, 6, and 8 h treatments, images of pancreatic acinar cells were captured (4 images / well / time point) by Axio Observer 7 (Carl Zeiss, Oberkochen, Germany) fluorescent microscope with Zen 2.6 Pro software. In the case of PI, the excitation/emission wavelengths were 533-558/570-645, while for H33342 these were 365/420-470. ImageJ software was employed to measure the area of PI and H33342 stainings. Then the percent of PI was calculated by dividing the areas of PI and H33342. Triton X-100 treatment was considered as resulting in maximum toxicity.

### Measurement of reactive oxygen species

The intracellular ROS was determined by a microfluorescence method. Isolated pancreatic acinar cells were loaded with 10 µM 6-carboxy-2',7'-dichlorodihydrofluorescein diacetate (carboxy-H_2_DCFDA) for 30 min at 37 °C, then cells were washed with HEPES solution (140 mM NaCl, 5 mM KCl, 10 mM HEPES, 1 mM CaCl_2_, 1 mM MgCl_2_, 10 mM glucose; pH = 7.4) twice by centrifugation with 50 rcf at 4 °C for 2 min. Cells were resuspended in M199 medium without phenol red containing 2 mM L-glutamine, and 0.25 mg/ml soybean trypsin inhibitor. Cells were seeded into a 96-well plate and treated with DMTS (1, 3, 10, 20, 30, 60 µg/ml), menadione (10, 30, 50 µM), or with the combination of these agents. Then the plate was placed into a FLUOstar OPTIMA plate reader and every 8 min a measurement was performed for 2 h utilising 490 nm excitation and 544 nm emission. The fluorescence intensities were normalised to the untreated control group.

#### Real-time measurement of intracellular Ca^2+^ concentration

As the Ca^2+^ signalling is important in physiological and pathophysiological conditions of acinar cells, the ic[Ca^2+^] was followed in real-time. Acini were placed in standard HEPES solution and loaded with 5 µM FURA-2-AM (Biotium, Fremont, CA, USA) for 30–90 min in a humidified atmosphere at 37 °C containing 5% CO_2_. Then, the cells were placed on poly-L-lysine coated (5 µL from 0.01% w/v) coverslip and incubated for 20 min to allow cells to attach to the surface. Coverslips with cells were placed in Carl Zeis Axio Observer 7 microscope. Cells were perfused with standard HEPES and with various treatment solutions prepared in standard HEPES in the following order: (1) standard HEPES solution; (2) DMTS (0, 10, 20, 30 µg/ml); (3) 0.1 nM Caer or 1 nM Caer with or without 30 µg/ml DMTS; (4) DMTS (0 or 30 µg/ml); (5) standard HEPES solution; (6) 100 µM carbachol. The perfusion rate was 4–6 ml/min. Five to fifteen small areas (regions of interest) of 3–10 cells in each acinus intact were monitored. The cells were excited with light at wavelengths of 340 and 380 nm, and the 340/380 fluorescence excitation ratio was measured at 510 nm. One ic[Ca^2+^] measurement was obtained per second. For the quantitative assessment of Ca^2+^ responses, the number (shown as frequency) and height (shown as calcium response) of individual spikes recorded between 480 and 960 s (8 min) were counted and averaged to be presented as mean ± standard deviation (SD). In case of supramaximal (1 nM) Caer administration the area under the curve (AUC) and the decreasing slope of ic[Ca^2+^] response were calculated.

#### LC–MS/MS measurement of low molecular weight (LMW) metabolites from pancreatic tissue and serum samples

Measurements were based on the method published by Akaike et al^81^. The following low molecular weight (LMW) metabolites were determined: cysteine, cysteine persulfide, glutathione, glutathione persulfide, sulfide, lanthionine, homolanthionine, cystine, cystathionine. Briefly, frozen pancreatic tissue was homogenised with a dismembrator, then resuspended in ice-cold methanol containing 5 mM β-(4-hydroxyphenyl)ethyl iodoacetamide (HPE-IAM). After sonication, the derivatisation was performed at 37ºC for 20 min and followed by a centrifugation step (14,000 g; 10 min; 4ºC). Then, 100 µl of the supernatant was acidified with 10% formic acid (FA) and diluted two-fold with 0.1% FA/H_2_O before injection. Tissue pellets were dissolved in 1% SDS in phosphate-buffered saline, and following sonication protein content was measured using a BCA assay (Pierce BCA Protein Assay Kit, Thermo Fisher Scientific, Waltham, MA, USA).

75 µl of ice-cold methanol containing 5 mM HPE-IAM was added to 25 µl of serum sample. Then the mixtures were incubated at 37ºC for 20 min and centrifuged at 14,000 g; 10 min; 4ºC. The supernatant was acidified with 10% FA and diluted two-fold with 0.1% FA/H_2_O before injection.

Liquid chromatography-tandem mass spectrometry (LC–MS/MS) measurements were carried out on a Thermo Q-Exactive Focus Orbitrap mass spectrometer coupled to a Thermo Vanquish UHPLC (ultra-high-performance liquid chromatograph) and the samples were analyzed with two different methods. MS/MS detection was conductedin positive ionisation mode, and higher-energy collisional dissociation was used to detect the analytes detailed in Supplementary Table 1.

Measurement of derivatised analytes was conducted on a Phenomenex Kinetex C18 (50 × 2.1 mm, 2.6 µm) column with eluents 0.1% FA/H_2_O (A) and 0.1% FA/MeOH (B). The initial 5% B was linearly increased first to 13% in 2 min, then to 95% in 4 min, held there for 0.5 min, then lowered back to 5% B in 0.1 min and held there for 3.4 min before the next injection. The flow rate was 0.5 ml/min, the column was thermostated at 40 °C.

A Thermo Scientific Hypercarb (100 × 2.1 mm, 3 µm) column was used to detect thioeter species with eluents of 0.5% FA/H_2_O (A) and 0.5% isopropyl alcohol:acetonitrile 1:1 (B). Initial 0% B was linearly increased to 30% in 15 min, then to 100% in 1 min, kept for 5 min, then decreased to 0% B in 1 min, held for 8 min. The column temperature was 40 °C and the flow rate was 0.2 ml/min.

#### RNAscope in situ hybridisation combined with immunofluorescence

Pancreatic tissues from male FVB/n mice were collected. Samples were fixed in 4% paraformaldehyde for 36 h at 4°C. Paraffin embedding was carried out following standard procedure. Five μm thick sections were cut and mounted.

RNAscope in situ hybridisation (ISH) was performed to detect the expression of *Trpa1.* Urocortin3 (*Ucn3*) mRNA was applied as a marker of pancreatic Langerhans islets. The pretreatment and the RNAscope ISH (probe hybridisation, signal amplification and channel development) were performed according to RNAscope Multiplex Fluorescent Reagent Kit v2 user manual (ACD, Hayward, CA, USA). Mouse *Trpa1* (ACD; cat.no.: 400211) and *Ucn3* (464,861-C3) were visualised by cyanine 3 (Cy3) (1:750 for *Trpa1*) and cyanine 5 (Cy5) (1:750 for *Ucn3*) dyes, respectively.

Following the RNAscope procedure, slides were treated with an anti-amylase mouse antibody (cat.no.: sc-514229; Santa Cruz Biotechnology, TX, USA) (1:250) used as an exocrine acinar marker for 24 h at 24°C. After 2 × 15 min washes, slides were treated with Alexa 488-conjugated donkey anti-mouse antibody (cat.no.: 715-545-150; Jackson Immunoresearch,) for 3 h at 24°C. Sections were counterstained with DAPI (ACD) and were covered with ProLong Gold Antifade (Thermo Fisher Scientific, Waltham, MA, USA) mounting medium.

Mouse 3-plex positive (ACD; cat.no: 320881) control probes specific to *Polr2a*, *Ppib* and *Ubc* mRNA and 3-plex negative (ACD; cat.no: 320871) control probes to bacterial *dabP* mRNA were tested. The 3-plex positive control probes provided a well-detectable signal in the pancreas, while the negative control probes did not offer any recognisable fluorescence in the preparations (images not presented).

*Microscopy and digital imaging*: Fluorescently labelled sections were digitalised using an Olympus FluoView 1000 confocal microscope (Olympus, Europa, Hamburg, Germany) in sequential scanning in analogue mode. We tilised an 80 µm confocal aperture (optical thickness 3.5 µm), 1024 × 1024 pixel resolution, and a 60 × objective for scanning. The excitation and emission spectra for the respective fluorophores were selected using built-in settings of the FluoView software (FV10-ASW; Version 0102, Olympus, Europa, Hamburg, Germany). DAPI was excited at 405 nm, Cy3 at 550 nm, Alexa 488 at 488 nm and Cy5 at 647 nm. Sections were scanned for the respective wavelengths at four channels.

#### Statistical analysis

The sample size calculation was performed prior to in vivo studies with G ∗ Power^82^ (effect size: 1.8; power: 0.95; alpha error probe: 0.05). Data are presented as means ± SD. Experiments were evaluated by student’s t-test, and one- or two-way ANOVA followed by Tukey, Dunnett or Dunn *post-hoc* tests (JASP, JASP Team (2022), version 0.16.3). *P* < 0.05 was accepted as statistically significant. All means, SD, and sample numbers are shown in Supplementary Table 1.

### Supplementary Information

Supplementary Information 1.

Supplementary Information 2.

## Data Availability

The datasets generated and/or analysed during the current study are available from the corresponding authors on reasonable request.

## Abbreviations

Ach: Acetylcholine
AP: Acute pancreatitis
Caer: Caerulein
CBS: Cystathionine β-synthase
CDC: Chenodeoxycholate
CGRP: Calcitonin gene-related peptide
CSE: Cystathionine -γ-lyase
DMSO: Dimethyl sulfoxide
DMTS: Dimethyl trisulfide
EtOH-POA: Ethanol and palmitoleic acid
GAPDH: Glyceraldehyde-3-phosphate dehydrogenase
H_2_O_2_: Hydrogen peroxide
H_2_S: Hydrogen sulfide
ic[Ca^2+^]: Intracellular Ca^2+^ concentration
LC–MS/MS: Liquid chromatography-tandem mass spectrometry
L-Arg: L-arginine-HCl
LO: L-ornithine-HCl
MPO: Myeloperoxidase
MTT: 3-(4,5-Dimethylthiazol-2-yl)-2,5-diphenyltetrazolium bromide
NF-κB: Nuclear factor κB
Nrf2: Nuclear factor-erythroid 2 related factor 2
PI: Propidium iodide
Poly80: Polysorbate 80
PS: Physiological saline
ROS: Reactive oxygen species
SDS: Sodium dodecyl sulfate
TNF-α: Tumour necrosis factor-α
TRPA: Transient receptor potential ankyrin
