# Supplementary material for: The anti-inflammatory effect of dimethyl trisulfide in experimental acute pancreatitis
Source: Sci Rep. 2023 Oct 5;13:16813. doi: 10.1038/s41598-023-43692-9 (PMC10556037; doi:10.1038/s41598-023-43692-9)
Supplement: Supplementary file 1 — Supplementary Information 1. [file 41598_2023_43692_MOESM1_ESM.docx]

**The anti-inflammatory effect of dimethyl trisulfide in experimental acute pancreatitis**

Erik Márk Orján^1^, Eszter Sára Kormányos^1^, Gabriella Mihalekné Fűr^1^, Ágnes Dombi^2^, Emese Réka Bálint^1^, Zsolt Balla^1^, Beáta Adél Balogh^1^, Ágnes Dágó^1^, Ahmad Totonji^1^, Zoárd István Bátai^2^, Eszter Petra Jurányi^3,4^, Tamás Ditrói^3^, Ammar Al-Omari^2^, Gábor Pozsgai^2^, Viktória Kormos^2^, Péter Nagy^3,5,6^, Erika Pintér^2^, Zoltán Rakonczay Jr^1#^, Lóránd Kiss^1#^

# **Supplementary figures**

| 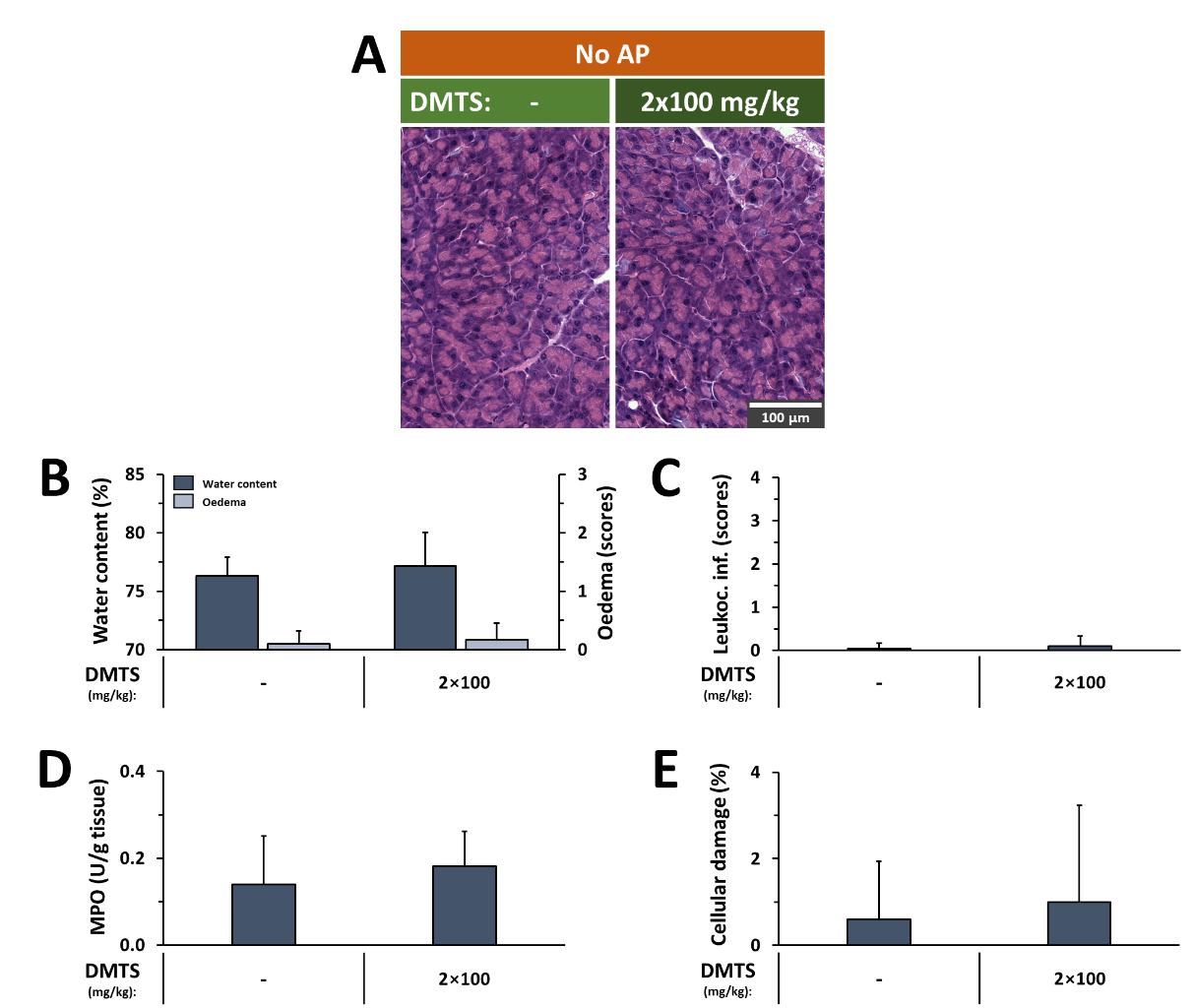 |
| --- |
| **Figure S1.** Two times dimethyl trisulfide (DMTS) treatment alone had no effect on the pancreas compared to control animals. Mice were treated with 2 × 100 mg/kg DMTS subcutaneously. Control animals received physiological saline instead of DMTS. Animals were sacrificed at 12 h after the first physiological saline injection. (A) Representative histopathological images of pancreatic tissues of the treatment groups. Bar charts show the extent of pancreatic (B) water content (as measured by the dry-wet weight ratio) and oedema (evaluation of histological sections), (C) leukocyte infiltration, (D) myeloperoxidase (MPO) activity, and (E) cellular damage. Values represent means with standard deviation (SD). The total number of animals was 12, for details of exact means, SDs, and animals per group please view Supplementary Table 1. Independent Samples T-Test was performed. Abbreviation: Leukoc. inf., leukocyte infiltration. |

| 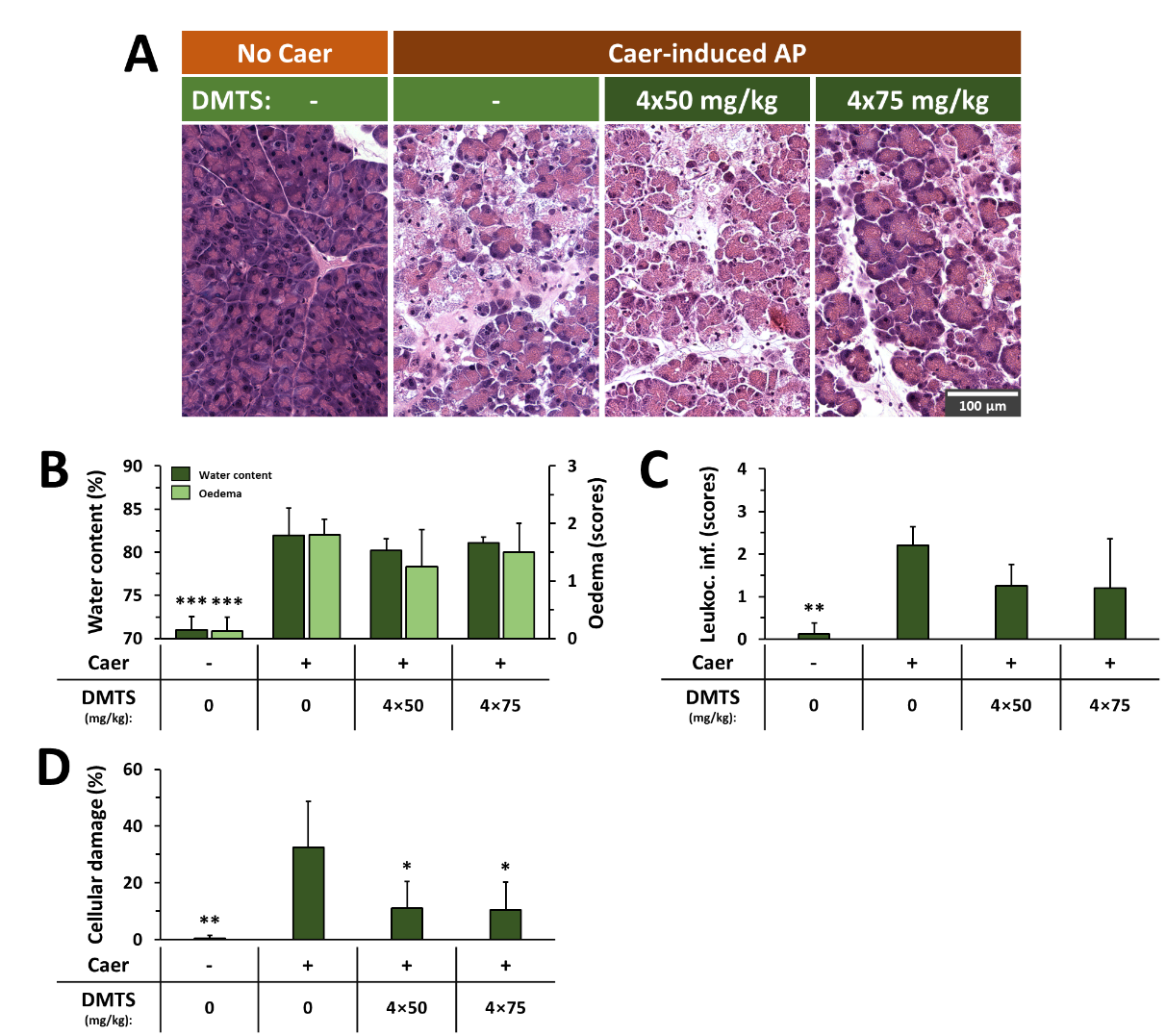 |
| --- |
| **Figure S2.** Dimethyl trisulfide (DMTS) administration for four times reduces the severity of caerulein (Caer)-induced necrotizing acute pancreatitis (AP) in mice. Animals were treated with 4 × 50 or 4 × 75 mg/kg DMTS subcutaneously, while intraperitoneal injection with 10 × 50 µg/kg Caer was used to induce AP. Control animals received physiological saline instead of Caer and vehicle instead of DMTS. Animals were sacrificed at 12 h after the first Caer or physiological saline injection. (A) Representative histopathological images of pancreatic tissues of the treatment groups. Bar charts show the extent of pancreatic (B) water content (as measured by the dry-wet weight ratio) and oedema (evaluation of histological sections), (C) leukocyte infiltration, and (D) cellular damage. Values represent means with standard deviation (SD). The total number of animals was 24, for details of exact means, SDs, and animals per group please view Supplementary Table 1. One-way ANOVA was performed followed by Dunnett’s post-hoc test where all of the groups were compared to the AP only group, *p < 0.05; **p < 0.01; ***p < 0.001. |

| 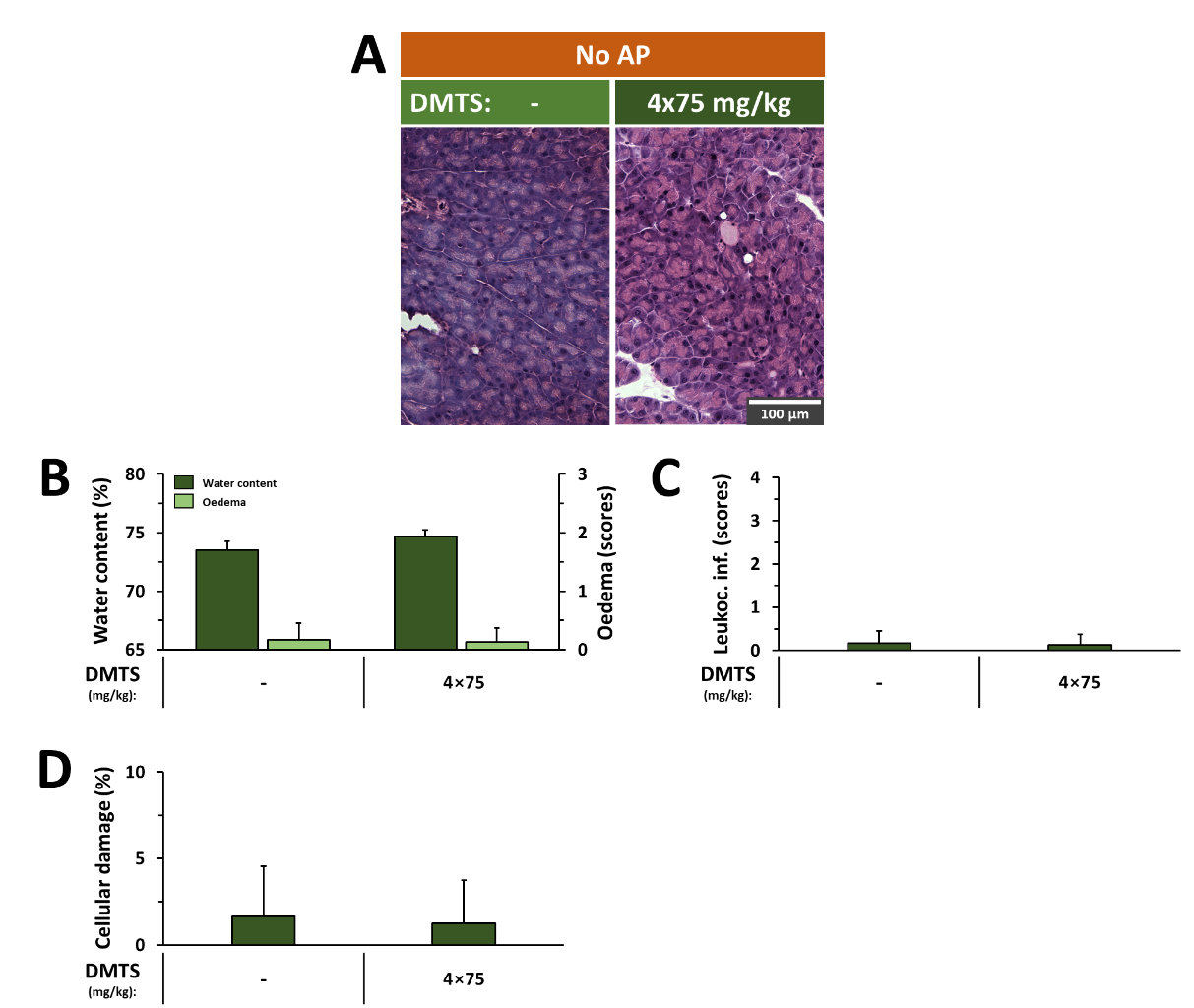 |
| --- |
| **Figure S3.** Four times dimethyl trisulfide (DMTS) treatment alone had no effect on the pancreas compared to control animals. Mice were treated with 4 × 75 mg/kg DMTS subcutaneously. Control animals received physiological saline instead of DMTS. Animals were sacrificed at 12 h after the first physiological saline injection. (A) Representative histopathological images of pancreatic tissues of the treatment groups. Bar charts show the extent of pancreatic (B) water content (as measured by the dry-wet weight ratio) and oedema (evaluation of histological sections), (C) leukocyte infiltration, and (D) cellular damage. Values represent means with standard deviation (SD). The total number of animals was 12, for details of exact means, SDs, and animals per group please view Supplementary Table 1. Independent Samples T-Test was performed. |

| 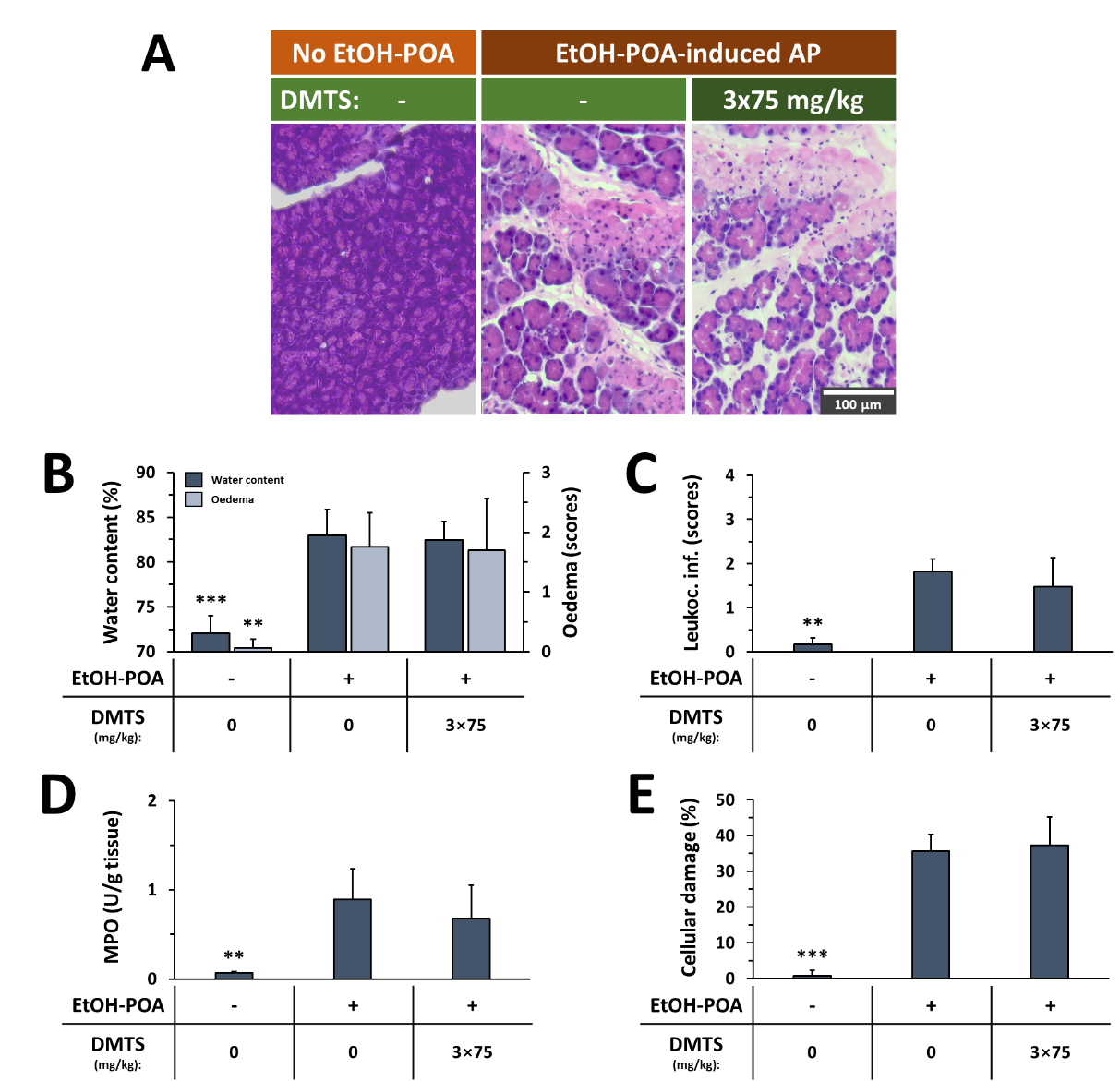 |
| --- |
| **Figure S4.** Dimethyl trisulfide (DMTS) administration at 0, 3, and 12 hours was unable to reduce the severity of acute pancreatitis (AP). Mice were treated subcutaneously with 3 × 75 mg/kg DMTS, whereas intraperitoneal injection with 2 × 1.35 g/kg ethanol (EtOH) and 2 × 150 mg/kg palmitoleic acid (POA) were used to induce AP. Control animals received physiological saline instead of EtOH-POA, or vehicle instead of DMTS. Animals were sacrificed 24 h after the first injection of EtOH-POA or physiological saline injection. (A) Representative histopathological images of pancreatic tissues of the treatment groups. Bar charts show the extent of pancreatic (B) water content (as measured by the dry-wet weight ratio) and oedema (evaluation of histological sections), (C) leukocyte infiltration, (D) myeloperoxidase (MPO) activity, and (E) cellular damage. Values represent means with standard deviation (SD). The total number of animals was 20, for details of exact means, SDs, and animals per group please view Supplementary Table 1. (B, D–E) One-way ANOVA was performed followed by Dunnett’s post-hoc test where all of the groups were compared to the EtOH-POA only group, **p < 0.01; ***p < 0.001. (C) Kruskal-Wallis test was performed followed by Dunn’s post-hoc test, the groups were compared to the EtOH-POA only group, **p < 0.01. |

| 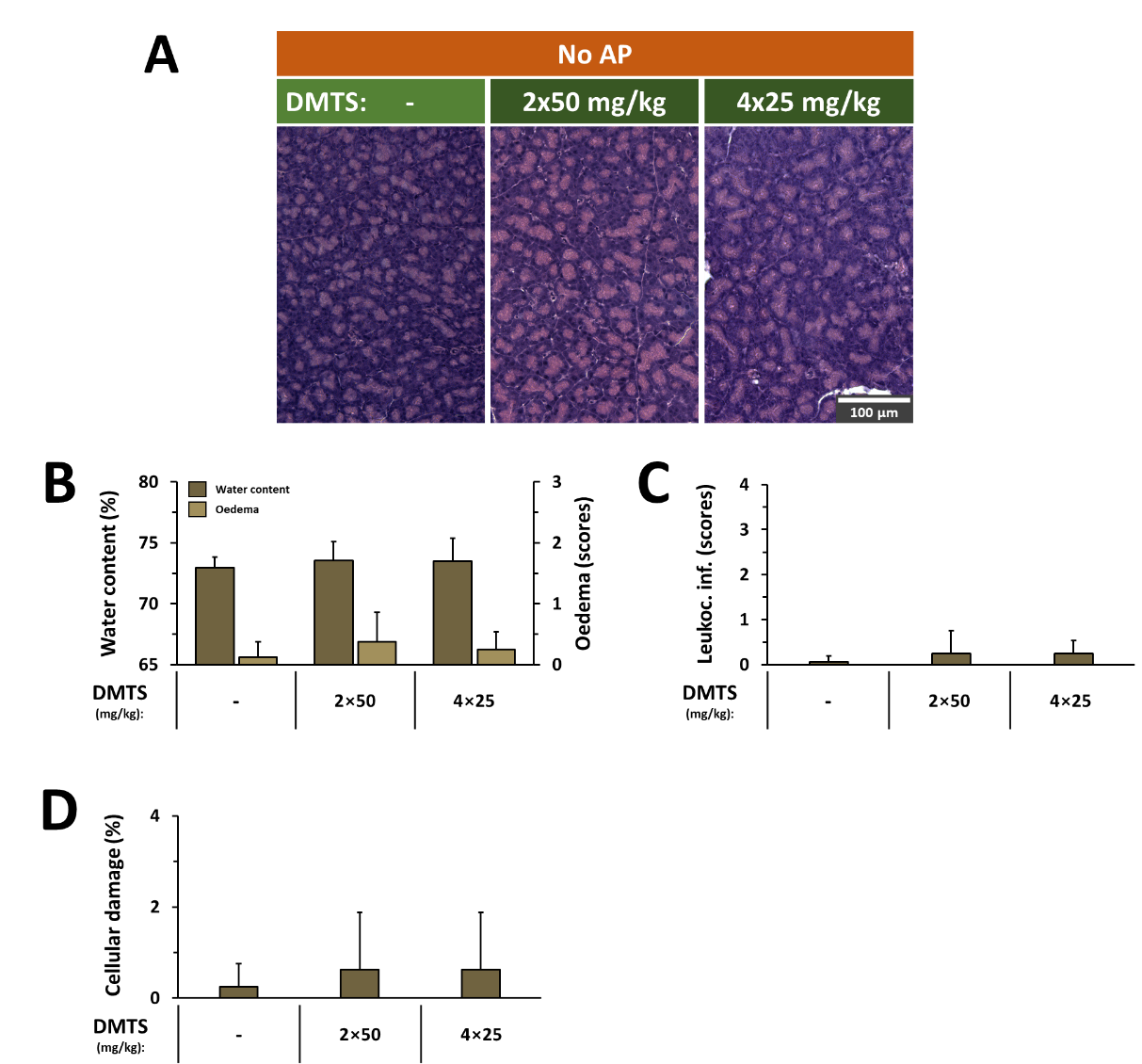 |
| --- |
| **Figure S5.** Dimethyl trisulfide (DMTS) treatment alone had no effect on the pancreas compared to control animals. Rats were treated with 2 × 50 or 4 × 25 mg/kg DMTS subcutaneously. Control animals received physiological saline instead of DMTS. Animals were sacrificed at 24 h after the first DMTS or physiological saline injection. (A) Representative histopathological images of pancreatic tissues of the treatment groups. Bar charts show the extent of pancreatic (B) water content (as measured by the dry-wet weight ratio) and oedema (evaluation of histological sections), (C) leukocyte infiltration, and (D) cellular damage. Values represent means with standard deviation (SD). The total number of animals was 18, for details of exact means, SDs, and animals per group please view Supplementary Table 1. One-way ANOVA was performed followed by Dunnett’s post-hoc test where all of the groups were compared to the control group. |

| 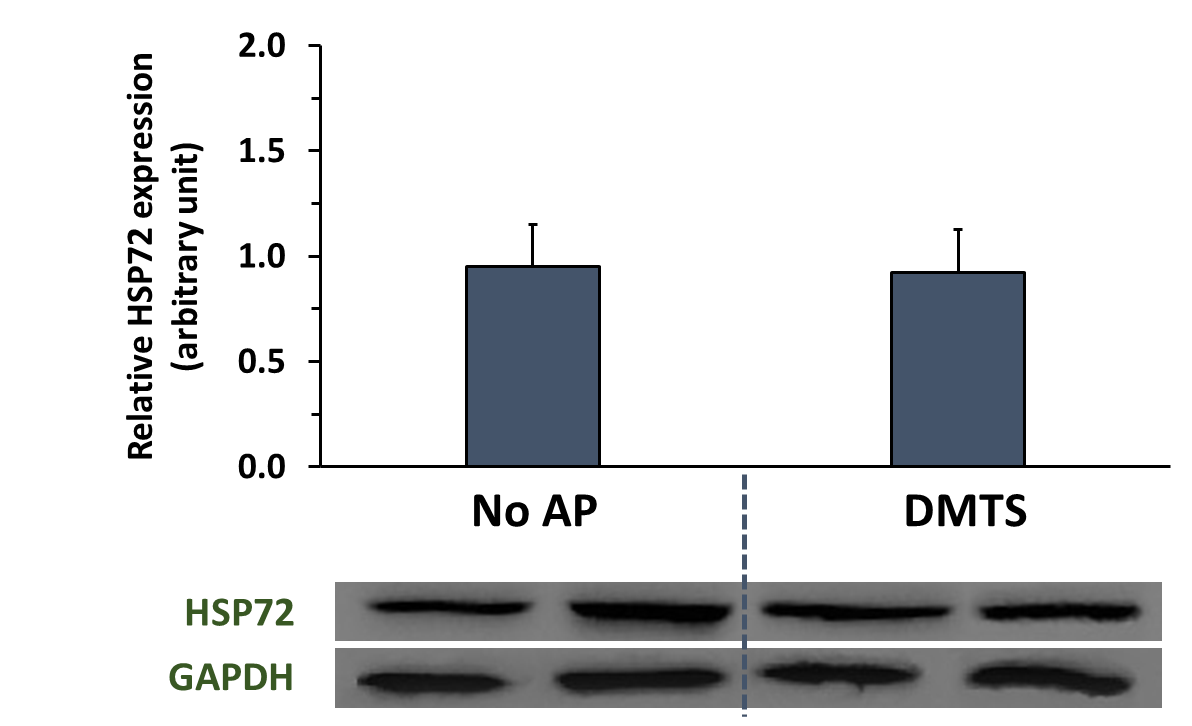 |
| --- |
| **Figure S6.** Changes in heat shock protein 72 (HSP72) expression during treatments with or without dimethyl trisulfide (DMTS) in mice without acute pancreatitis (AP). Representative Western blot image of pancreatic HSP72 expression, and the densitometry of Western Blot images for pancreatic HSP72 level. Band intensities were assessed by using ImageJ software and HSP72 expression was normalized to glyceraldehyde-3-phosphate dehydrogenase (GAPDH) levels. Fig. S12 provides full blot images. Values represent means with standard deviation (SD). The total number of animals was 8, for details of exact means, SDs, and animals per group please view Supplementary Table 1. One-way ANOVA was performed followed by Tukey’s post-hoc test. |

| 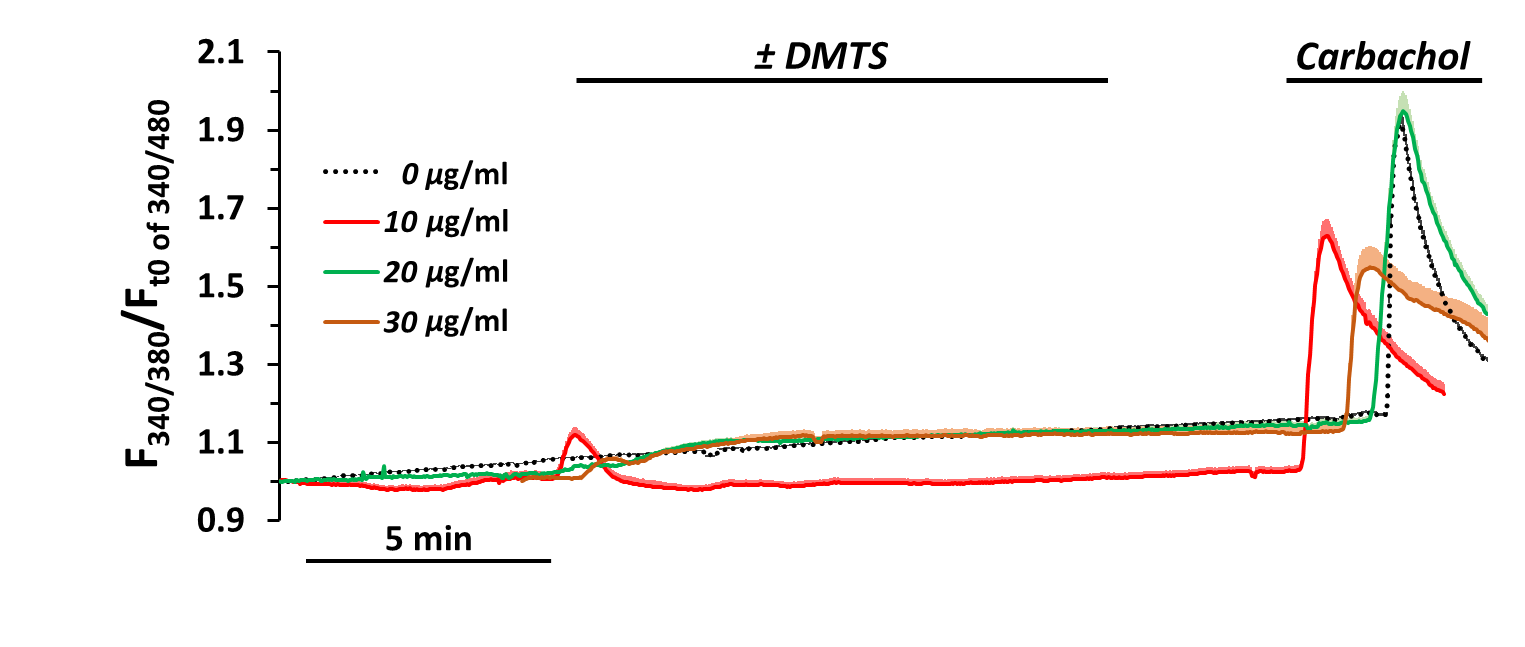 |
| --- |
| **Figure S7.** The effect of dimethyl trisulfide (DMTS) on intracellular Ca^2+^ concentration measured on isolated mouse pancreatic acinar cells. Representative traces of changes in intracellular Ca^2+^ concentrations in response to different DMTS treatments of isolated mouse pancreatic acinar cells are shown. Cells were perfused with standard HEPES solution and treated with 10, 20 or 30 µg/ml DMTS and 100 µM carbachol. |

|  |
| --- |
| **Figure S8.** Trpa1 mRNA expression in mouse pancreatic tissues. Exocrine glandular acini (A, B) show both Trpa1 mRNA (red) expression and amylase (green) immunofluorescence. Trpa1 transcripts (red) were also found in pancreatic ductal cells (C, D, yellow arrows). Urocortin3 (white) mRNA-expressing Langerhans islets (E, F) contain Trpa1 (red) transcripts. Scale bars: 10 µm in A–B and E–F; 20 µm in C–D. DAPI (blue) nuclear counterstain. |

| 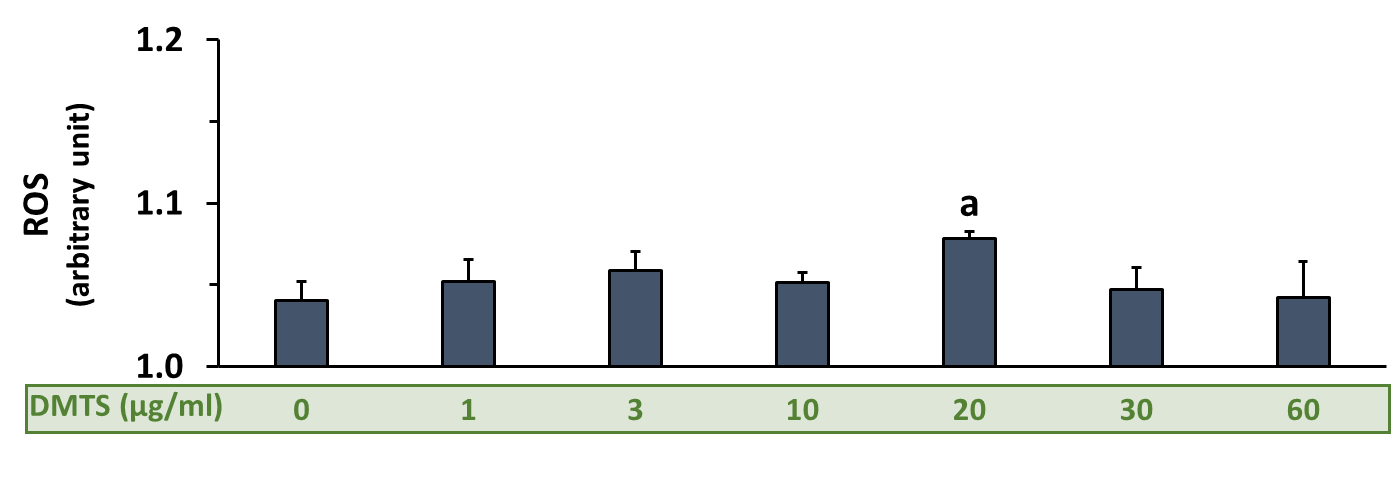 |
| --- |
| **Figure S9.** The effect of dimethyl trisulfide (DMTS) on reactive oxygen species (ROS) production in isolated mouse pancreatic acinar cells. DMTS treatment at 1, 3. 10, 20, 30 and 60 µg/ml. Values represent means with standard deviation (SD). 5 parallels were used (a total of 35 wells), and the cells were derived from 2 different animals. For details of exact means and SDs please view Supplementary Table 1. One-way ANOVA was performed followed by Tukey’s post-hoc test. Statistically signiﬁcant differences were marked in the following manner: ‘a’, vs. control (p < 0.05). |

| 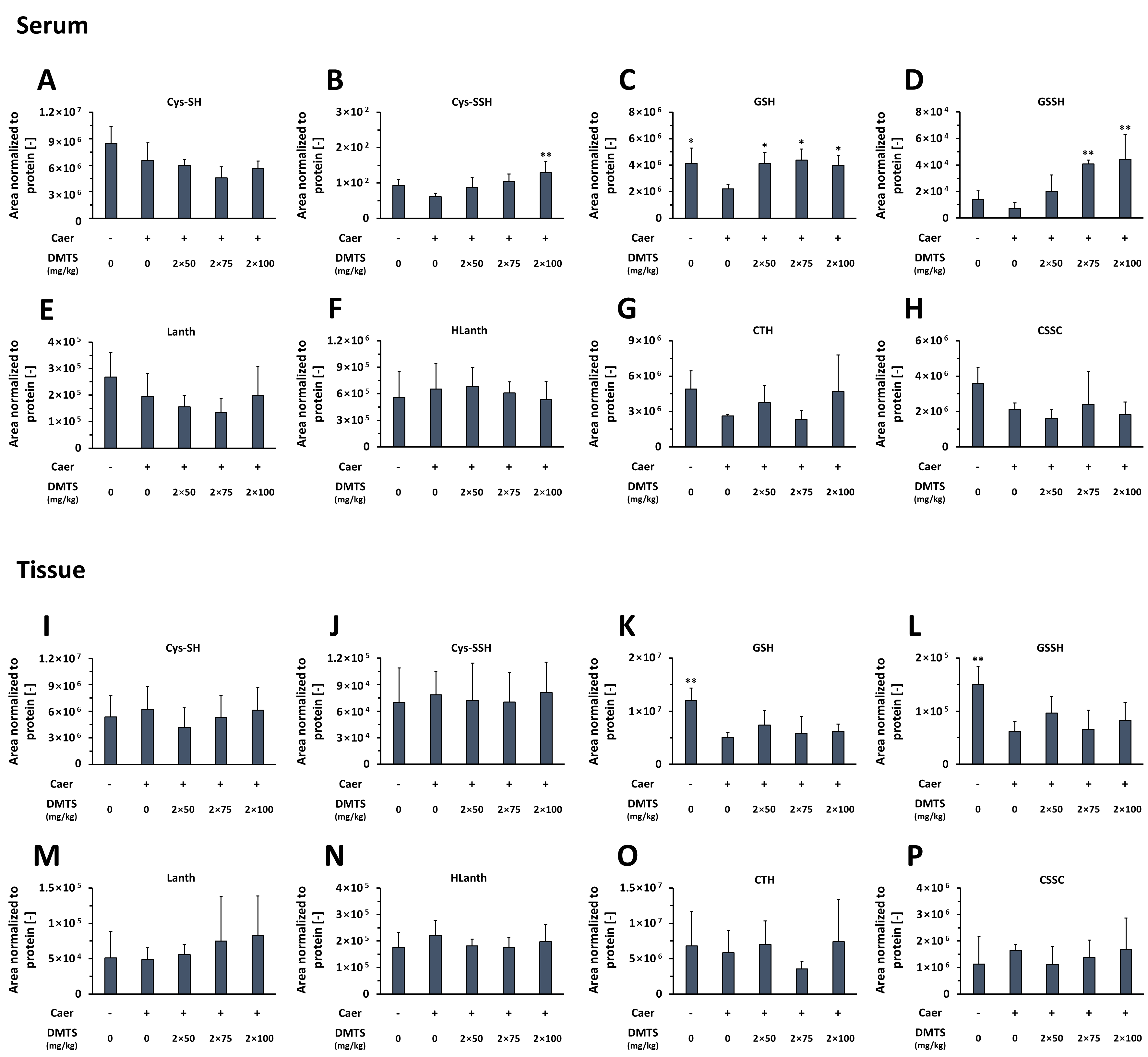 |
| --- |
| **Figure S10.** *Quantitative assessment of the effect of dimethyl trisulfide (DMTS) on sulfur species in acute pancreatitis (AP). Sulfur metabolome analyses of cysteine (Cys-SH), glutathione (GSH), lanthionine (Lanth), homolanthionine (HLanth), cystathionine (CTH) and cystine (CSSC) levels in serum (A, C, E–H) and tissue samples (I, K, M–P), respectively. The bar charts show Cys-SH and GSH persulfidation (Cys-SSH and GSSH) levels in serum (B, D) and tissue samples (J, L), respectively. Values represent means with standard deviation (SD). The total number of animals was 22, for details of exact means, SDs, and animals per group please view Supplementary Table 1. (A–D, F–P) One-way ANOVA was performed followed by Dunnett’s post-hoc test where all of the groups were compared to the Caer only group, *p < 0.05; **p < 0.01. (E) Kruskal-Wallis test was performed followed by Dunn’s post-hoc test, the groups were compared to the Caer only group, **p < 0.01.* |

**Figure S11.** *Quantitative assessment of pancreatic tumor necrosis factor α (TNF-α) and interleukin 1β (IL-1β) expression in a mouse model of caerulein-induced (6 or 10 × 50 µg/kg intraperitoneal hourly injections) acute pancreatitis. The cytokines were analysed at different time points (0, 6, 10, 12, 24, 48 hours) during progression of the disease. The protein content in each sample was 0.1 mg/ml. Values represent means with standard deviation (SD). The total number of animals was 30, for details of exact means, SDs, and animals per group please view Supplementary Table 1. One-way ANOVA was carried out followed by Dunnett’s post-hoc test where all of the groups were compared to the 0h group,*p < 0.05; **p < 0.01.*

| 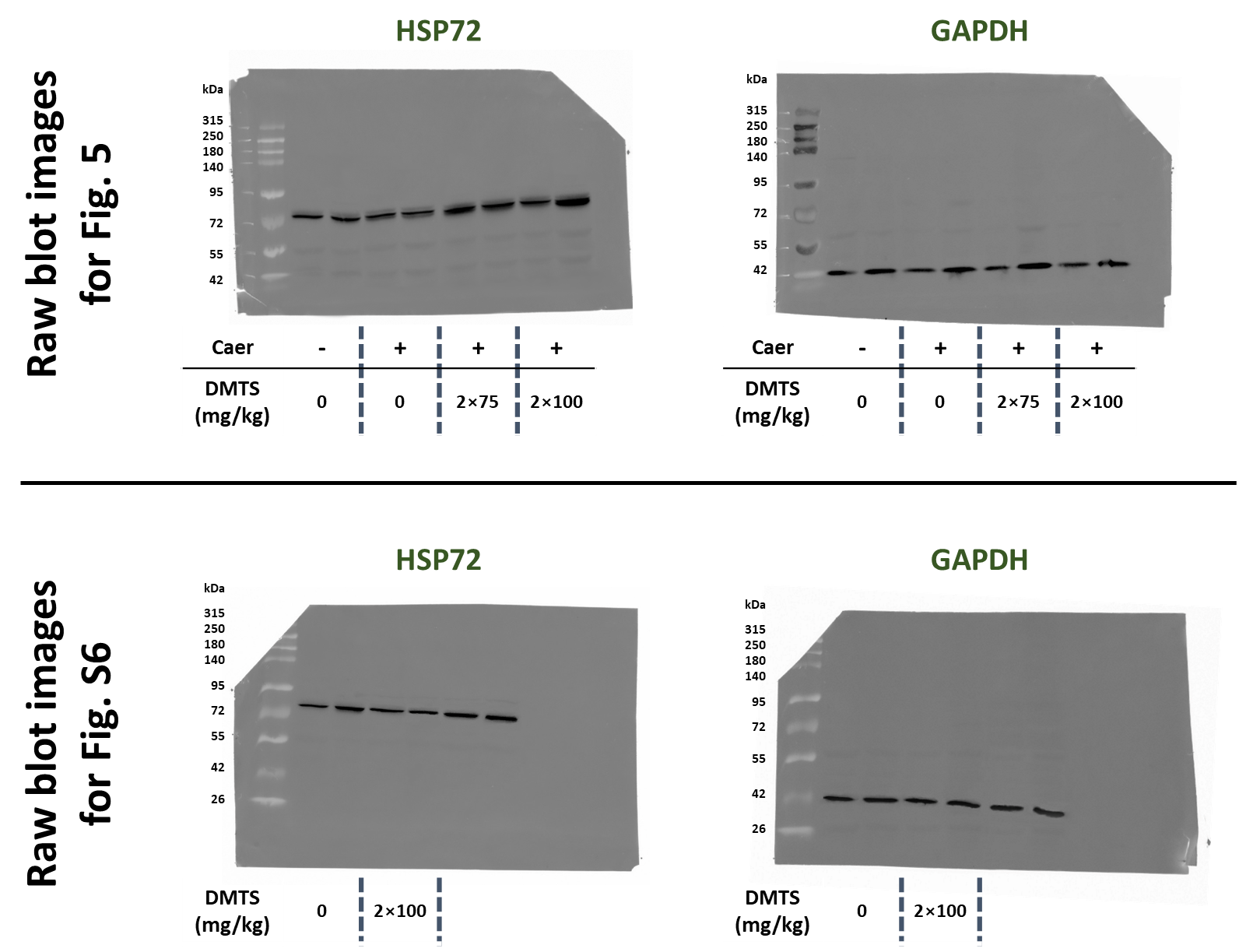 |
| --- |
| **Figure S12.** *Original full-length Western blot images. All samples were derived from the same experiment and all blots were processed in parallel.* |

# **Supplementary Methods**

## Measurement of pancreatic TNF-α and IL-1β expression at different time points of Caer-induced AP

Mice were injected with Caer (6 or 10 × 50 µg/kg i.p.) and were sacrificed at 0 (control animals), 6, 10, 12, 24, 48 h. The pancreata were collected and were frozen at –80°C until measurement. Then the tissues were homogenized in homogenization buffer (10mM Na-HEPES, 1µM MgCl_2_, 10mM KCl, 5mM iodoacetamide, 4mM benzamidine-HCl, 1mM DL-Dithiothreitol, 1mM Phenylmethyl sulfonylfluoride) by using 3 × 15 s sonication. Following incubation (20 min at 0°C), the homogenates were centrifuged at 20000 rcf at 4°C for 20 min and supernatants were kept for further measurements. The protein concentration of the pancreatic tissues were measured by the Bradford method, and each sample was diluted to 0.1 mg/ml protein concentration. The TNF-α and IL-1β concentrations were measured by a commercial ELISA kit from R&D Systems (Minneapolis, MN, USA) as described by the manufacturer.
